# PIM-induced phosphorylation of Notch3 promotes breast cancer tumorigenicity in a CSL-independent fashion

**DOI:** 10.1016/j.jbc.2021.100593

**Published:** 2021-03-26

**Authors:** Sebastian K.J. Landor, Niina M. Santio, William B. Eccleshall, Valeriy M. Paramonov, Ellen K. Gagliani, Daniel Hall, Shao-Bo Jin, Käthe M. Dahlström, Tiina A. Salminen, Adolfo Rivero-Müller, Urban Lendahl, Rhett A. Kovall, Päivi J. Koskinen, Cecilia Sahlgren

**Affiliations:** 1 Faculty of Science and Engineering/Cell Biology, Åbo Akademi University, Turku, Finland; 2 Turku Bioscience, University of Turku and Åbo Akademi University, Turku, Finland; 3 Department of Biology, University of Turku, Turku, Finland; 4 Institute of Biomedicine, Research Centre for Integrative Physiology and Pharmacology, University of Turku, Turku, Finland; 5 Department of Molecular Genetics, Biochemistry, and Microbiology, University of Cincinnati, Ohio, USA; 6 Department of Cell and Molecular Biology, Karolinska Institute, Stockholm, Sweden; 7 Structural Bioinformatics Laboratory, Biochemistry, Faculty of Science and Engineering, Åbo Akademi, Turku, Finland; 8 Department of Biomedical Engineering, Institute for Complex Molecular Systems, Eindhoven University of Technology, Eindhoven, The Netherlands

**Keywords:** PIM kinases, Notch proteins, phosphorylation, protein–protein interaction, transcription regulation, tumor cell biology, breast cancer, CAM, chorioallantoic membrane, CHX, cycloheximide, CSL, C promoter–binding factor 1, Suppressor of Hairless, Lag-1, DHPCC-9, 1,10-dihydropyrrolo[2,3-a]carbazole-3-carbaldehyde, DLL, Delta-like ligand, DMSO, dimethyl sulfoxide, ER, estrogen receptor, GFP, green fluorescent protein, HES1, hairy and enhancer of split 1, HEY1, hairy/enhancer-of-split related with YRPW motif protein 1, ICD, intracellular domain, ITC, isothermal titration calorimetry, KI, knock-in, NICD, Notch intracellular domain, N1ICD, Notch1 ICD, N3ICD, Notch3 ICD, N3RAM, NOTCH3 RAM, PAS, phospho-AKT substrate, PDB, Protein Data Bank, RAM, RBPJ-associated molecule, RBPJ, recombination signal–binding protein for immunoglobulin kappa J region, RFP, red fluorescent protein, SA, serine to alanine, SE, serine to glutamic acid

## Abstract

Dysregulation of the developmentally important Notch signaling pathway is implicated in several types of cancer, including breast cancer. However, the specific roles and regulation of the four different Notch receptors have remained elusive. We have previously reported that the oncogenic PIM kinases phosphorylate Notch1 and Notch3. Phosphorylation of Notch1 within the second nuclear localization sequence of its intracellular domain (ICD) enhances its transcriptional activity and tumorigenicity. In this study, we analyzed Notch3 phosphorylation and its functional impact. Unexpectedly, we observed that the PIM target sites are not conserved between Notch1 and Notch3. Notch3 ICD (N3ICD) is phosphorylated within a domain, which is essential for formation of a transcriptionally active complex with the DNA-binding protein CSL. Through molecular modeling, X-ray crystallography, and isothermal titration calorimetry, we demonstrate that phosphorylation of N3ICD sterically hinders its interaction with CSL and thereby inhibits its CSL-dependent transcriptional activity. Surprisingly however, phosphorylated N3ICD still maintains tumorigenic potential in breast cancer cells under estrogenic conditions, which support PIM expression. Taken together, our data indicate that PIM kinases modulate the signaling output of different Notch paralogs by targeting distinct protein domains and thereby promote breast cancer tumorigenesis *via* both CSL-dependent and CSL-independent mechanisms.

The Notch signaling pathway orchestrates tissue development and homeostasis, but when dysregulated, it can also promote tumorigenesis and support cancer progression (1, 2, 3, 4). The Notch pathway relies on cell–cell contacts, where membrane-spanning Jagged and Delta-like ligands (DLLs) on signal-sending cells bind to Notch receptors on signal-receiving cells. This induces two sequential receptor cleavages, releasing the Notch intracellular domain (NICD), which translocates to the nucleus and forms a transcriptionally active complex with the Mastermind-like transcriptional coactivator and CSL (C promoter–binding factor 1, Suppressor of Hairless, Lag-1), also known as RBPJ (recombination signal–binding protein for immunoglobulin kappa J region). In addition to this canonical mode of Notch signaling, there are also alternative noncanonical signaling mechanisms, which are CSL-independent (1), although the functional consequences of these signaling mechanisms remain to be elucidated.

The ICDs of all four Notch receptor paralogs are composed of a CSL/RBPJ-associated molecule (RAM) domain, ankyrin repeats flanked by two nuclear localization sequences, a transactivation domain, and a C-terminal domain rich in proline, glutamic acid, serine, and threonine residues (2, 3). The stability and activities of NICDs are regulated by several types of post-translational modifications, such as phosphorylation, acetylation, hydroxylation, sumoylation, and ubiquitylation (4, 5, 6).

We have previously shown that the oncogenic PIM family kinases can phosphorylate Notch1 and Notch3 but not Notch2 (7). The serine-/threonine-specific PIM kinases were originally identified as proviral integration sites for Moloney murine leukemia virus and have since then been implicated in both hematological malignancies and solid cancers, where they support cancer cell proliferation, survival, metabolism, and motility by multiple mechanisms (8, 9, 10). PIM-induced phosphorylation of serine 2152 in mouse Notch1 (corresponding to human NOTCH1 S2162) is important for its nuclear localization as well as CSL-dependent transcriptional activity (7). Furthermore, this phosphorylation enhances the tumorigenic behavior of both breast and prostate cancer cells, as also suggested by the observed coexpression of *PIM1* and *NOTCH1* mRNAs in patients with breast cancer (7).

In this study, we have analyzed the regulation of Notch3 by PIM kinases. Unexpectedly, the PIM target sites are not conserved between Notch1 and Notch3, as PIM kinases phosphorylate serine 1673 in the RAM domain of mouse Notch3. Phosphorylated Notch3 ICD (N3ICD) cannot bind CSL to induce the canonical CSL-dependent transcriptional program. Surprisingly however, it supports *in vivo* tumor growth of breast cancer cell xenografts in the presence of estrogen. Thus, our data suggest that in the context of hormone-dependent breast cancer, PIM-mediated phosphorylation of Notch3 promotes tumor growth but *via* a different mechanism than phosphorylation of Notch1.

## Results

### Both Notch1 and Notch3 contribute to tumorigenic growth of breast cancer cells

**Figure 1:**
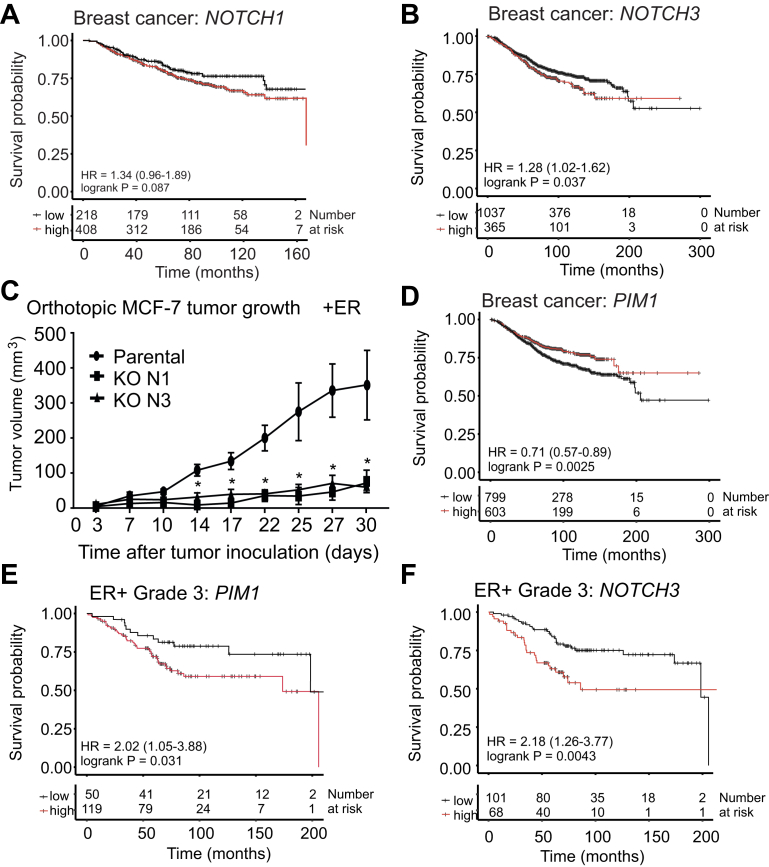
***NOTCH1* and *NOTCH3* promote breast carcinoma growth, whereas both *PIM1* and *NOTCH3* are connected to poor survival in ER-positive grade 3 cases.***A* and *B,* Kaplan–Meier analyses were performed to visualize the connection of *NOTCH1* and *NOTCH3* mRNA expression to overall survival in clinical breast cancer. *C,* parental MCF-7 cells or their KO derivatives were inoculated into mammary glands of athymic nude mice with previously implanted estradiol pellets. Tumor growth was followed by manual palpation. Shown are average tumor volumes at indicated time points. Error bars represent standard deviations. *D*–*F,* additional Kaplan–Meier analyses were performed on *PIM1* expression as well as the specified subgroups of clinical breast cancer data. Error bars represent standard deviations. ∗*p* < 0.05 was used as a limit for significant difference. ER, estrogen receptor; HR, hazard ratio.

In our previous study, we showed that PIM kinases phosphorylate Notch1 to promote tumorigenicity of estrogen receptor (ER)–positive breast cancer cells (7). In addition, we obtained preliminary evidence that also Notch3 may be a PIM substrate. To compare the clinical importance of NOTCH1 and NOTCH3 in breast cancer, Kaplan–Meier analyses were performed using an online breast cancer dataset (11). Interestingly, upregulation of both *NOTCH1* and *NOTCH3* mRNA was connected to poor survival, although only *NOTCH3* showed a statistically significant correlation (Fig. 1, *A* and *B*).

In order to evaluate the oncogenic potential of *NOTCH1* and *NOTCH3* on breast cancer growth *in vivo*, we used the CRISPR-/Cas9-based genomic editing to prepare MCF-7 breast cancer cell-based KO cell lines lacking endogenous expression of either *NOTCH1* or *NOTCH3* genes and the corresponding proteins (Fig. S1, *A*–*C*). WT or KO cells were orthotopically inoculated into the mammary glands of athymic nude mice. The parental MCF-7 cells efficiently formed tumors as expected, but the growth of tumors derived from the *NOTCH1* or *NOTCH3* KO cells was significantly decreased (Fig. 1*C*), indicating that both genes are essential for estrogen-dependent mammary tumorigenesis.

We were then interested in testing whether there is a similar protumorigenic cross talk between PIM1 and Notch3 as previously shown for Notch1 (7). Surprisingly, when analyzing the prognostic role of PIM1 by Kaplan–Meier analysis, we observed that in breast cancer cases in general, *PIM1* mRNA upregulation is rather protective (Fig. 1*D* and Table S1). However, when analyzing the ER-positive cases in more detail, we observed that in the most aggressive grade 3 cases, upregulation of both *PIM1* and *NOTCH3* mRNAs is correlated with poor survival (Fig. 1, *E* and *F*).

Additional analyses were performed using clinical breast cancer data derived from the PanCancer Atlas dataset (12). No major differences were detected between *PIM1* and *NOTCH3* mRNA expression levels in different breast cancer stages, whereas the *PIM1* levels were reduced in most subtypes as compared with control samples (Fig. S2, *A*–*D*). When the expression levels of *PIM1* and *NOTCH3* mRNA levels were compared with each other, positive correlations were observed in all stages, except stage IV (Fig. S2*E*). Moreover, a weak positive correlation was observed in the luminal A subtype but not in other subtypes (Table S1 and Fig. S2). Taken together, the clinical data prompted further investigations on the cross talk of PIM1 and N3ICD in ER-positive luminal A cells, such as MCF-7 cells.

### Mouse Notch3 is phosphorylated by PIM kinases at S1673

**Figure 2:**
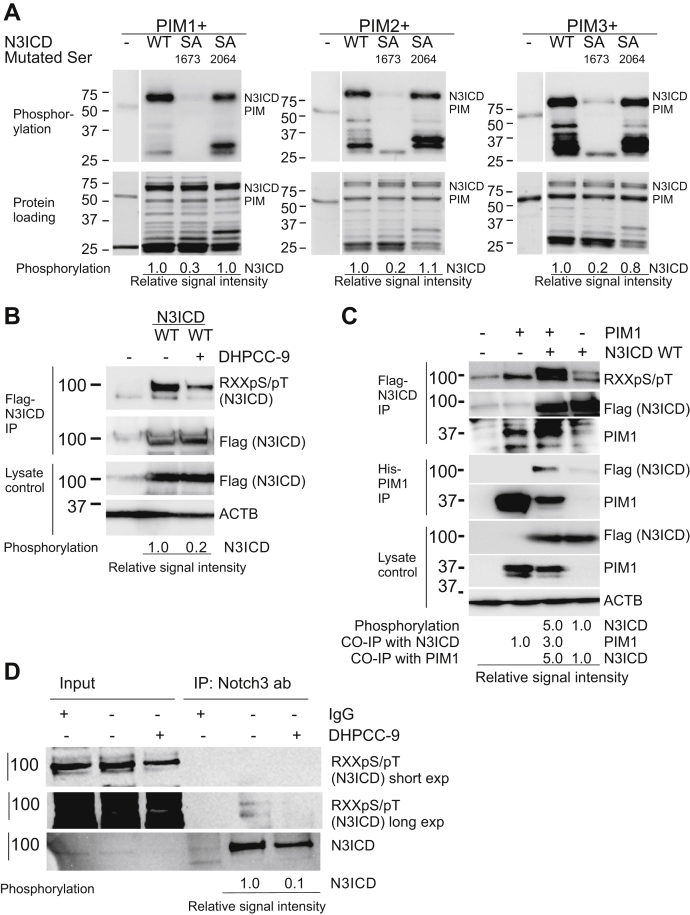
**PIM kinases phosphorylate mouse Notch3 ICD (N3ICD) at S1673.***A,* radioactive *in vitro* kinase assays were performed with GST-tagged PIM kinases and WT or serine to alanine (SA) mutants of mouse N3ICD. Phosphorylated proteins were separated by electrophoresis and detected by autoradiography. Protein loading was visualized by PageBlue staining. Shown are representative images from one of two experiments. *B* and *C,* MCF-7 cells were transiently transfected with plasmids encoding FLAG-tagged N3ICD, His-tagged PIM1, and/or their controls. About 24 h later, cells were treated with DMSO (−) or 10 μM DHPCC-9 (+). The ectopically expressed proteins were immunoprecipitated with the anti-FLAG affinity agarose gel or the HisLink resin. N3ICD phosphorylation was detected with the phosphospecific RXXpS/pT antibody and protein levels by anti-FLAG (N3ICD) or anti-PIM1 antibodies. Cell lysates and actin beta (ACTB) stainings were used as controls. Average signal intensities for phosphorylation or coimmunoprecipitation (CO-IP) were determined relative to overexpression levels. Shown are representative examples from three independent experiments. *D,* endogenous NOTCH3 was immunoprecipitated from MCF-7 cells cultured in the absence or the presence of DHPCC-9. N3ICD phosphorylation was detected with the phospho-AKT substrate antibody. Average signal intensities of phosphorylation were determined relative to overexpression levels. Shown are representative examples from three independent experiments.

Based on our previous results (7), we expected PIM kinases to phosphorylate mouse N3ICD at S2064, as this site (–KKSRRPPGK–) corresponds to the PIM target site S2152 (–KKARKPSTK–) in mouse Notch1. In addition, *in silico* analysis predicted another putative target site at S1673 (–RRKREHSTL–). To determine whether one or both serine residues are true PIM target sites, we used site-directed mutagenesis to replace them with alanine residues. When recombinant WT N3ICD protein or phosphodeficient serine to alanine (SA) mutants were subjected to radioactive *in vitro* kinase assays, the S2064A mutation did not reduce phosphorylation of N3ICD (Fig. 2*A*), indicating that S2064 is not a PIM target site. By contrast, all three PIM family members targeted S1673, as demonstrated by 70 to 80% decrease in phosphorylation of the S1673A mutant.

To confirm phosphorylation of Notch3 in a cellular context, FLAG-tagged N3ICD was transiently overexpressed in MCF-7 cells with or without His-tagged PIM1, and the samples were treated with the PIM-selective inhibitor 1,10-dihydropyrrolo[2,3-a]carbazole-3-carbaldehyde (DHPCC-9) or its solvent dimethyl sulfoxide (DMSO) as a control. The phosphorylation status of immunoprecipitated N3ICD was analyzed with the phospho-AKT substrate (PAS) antibody, which recognizes not only the AKT-targeted sequence RXXpS/pT but also the PIM-targeted consensus sequence RXRHXpS/pT (13). This analysis revealed that PIM inhibition decreases phosphorylation of N3ICD, whereas PIM1 overexpression increases it (Fig. 2*B*).

Coimmunoprecipitation of PIM1 and N3ICD also indicated that these proteins are able to interact in cells (Fig. 2*C*). To further demonstrate that endogenous N3ICD is phosphorylated in a PIM-dependent fashion, we immunoprecipitated NOTCH3 from MCF-7 cells cultured in the presence or the absence of the PIM inhibitor DHPCC-9. Analysis with the PAS antibody showed that PIM inhibition reduces the phosphorylation status of N3ICD (Fig. 2*D*), indicating that NOTCH3 is an endogenous PIM kinase target.

### PIM1 and N3ICD interact in breast cancer cells

**Figure 3:**
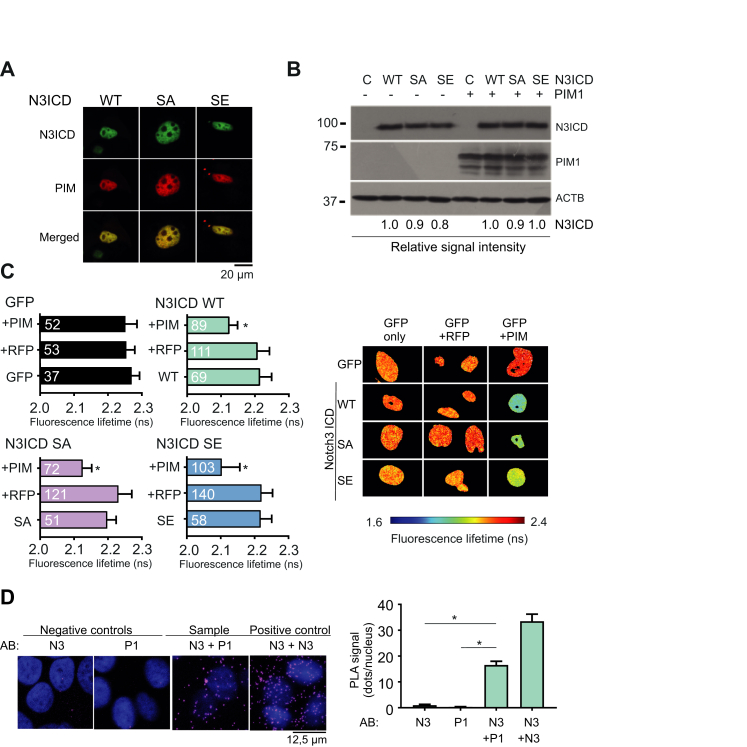
**PIM1 colocalizes and interacts with Notch3 ICD (N3ICD).** RFP-tagged PIM1 and/or GFP-tagged WT, phosphodeficient (SA), or phosphomimicking (SE) N3ICD were transiently overexpressed in MCF-7 cells. Empty vectors were used as controls. *A,* the localization of the fluorescent proteins was imaged by confocal microscopy. The figure shows representative single channel or merged images. *B,* equivalent expression levels of overexpressed N3ICDs were confirmed by Western blotting, and signal intensities were normalized to actin beta (ACTB) staining. *C,* the physical interactions between PIM1 and WT or mutant N3ICD were analyzed by fluorescence-lifetime imaging. The figure shows the results from three independent experiments along with sample numbers inside the *black bars* and representative images. *D,* proximity ligation assays were performed with one PIM1 and two different N3ICD antibodies (AB) to visualize the presence of endogenous proteins in the proximity of each other in MCF-7 cells. Representative images along with average results from three independent experiments are shown. ∗*p* < 0.05 was used as a limit for significant difference. Error bars represent standard deviation. SA, serine to alanine; SE, serine to glutamic acid; GFP, green fluorescent protein; RFP, red fluorescent protein.

We next used fluorescence microscopy to examine the interactions between fluorescently labeled forms of PIM1 and Notch3. N3ICD was tagged with green fluorescent protein (GFP), and the PIM-targeted S1673 residue was mutated to alanine to create a phosphodeficient mutant (SA) or to glutamic acid to create a phosphomimicking mutant (SE). When PIM1 tagged with red fluorescent protein (RFP) and GFP-tagged N3ICD were transiently overexpressed in MCF-7 cells and imaged by confocal microscopy, PIM1 colocalized to the same extent with the WT N3ICD as with the phosphomutants (Fig. 3*A*), indicating that they are likely to interact regardless of whether N3ICD is phosphorylated or not. Overexpressed PIM1 and N3ICD were predominantly localized in the nucleus irrespective of the phosphorylation status of N3ICD (Fig. 3, *A* and *B*). Fluorescence lifetime-imaging data confirmed the interactions of PIM1 with both the WT and mutant N3ICDs (Fig. 3*C*). However, when interactions of endogenously expressed human PIM1 and N3ICD proteins were analyzed by proximity ligation assays, strong positive signals were mostly detected in the cytoplasmic compartments (Fig. 3*D*), suggesting differences in the localization of overexpressed *versus* endogenous proteins.

### Phosphorylation of Notch3 by PIM1 inhibits binding to CSL

The PIM target residue in Notch3 is in the RAM domain, which is required for canonical Notch signaling activity as it mediates binding with the DNA-binding protein CSL (14, 15). To analyze the influence of phosphorylation on the interaction of the N3ICD RAM domain to CSL, we performed predictive molecular modeling, using the human Notch1 ICD (N1ICD) RAM and ankyrin repeat domains (16) as a template to model the corresponding areas in human N3ICD (Fig. S3). Minimization of the nonphosphorylated N3ICD caused only minor changes in the positions of amino acid side chains as compared with N1ICD. However, minimization of the N3ICD complex phosphorylated at S1672 (corresponding to mouse S1673) twisted the conformation of the NOTCH3 RAM (N3RAM) peptide, causing the phosphate group to point toward the solvent instead of interacting with CSL. This resulted in a slight displacement (root-mean-square deviation of 1.5 Å for Cα-atoms) of the phosphorylated N3RAM peptide as compared with the nonphosphorylated N3RAM. The binding site for S1672 resides in a small and hydrophobic cavity on CSL, suggesting that both the size and the negative charge on the phosphate group are likely to impair the binding of S1672 to CSL. In addition, the overall negative charge on the surface of CSL around the RAM-binding site is likely to repel the negative charge of the phosphorylated S1672.

**Figure 4:**
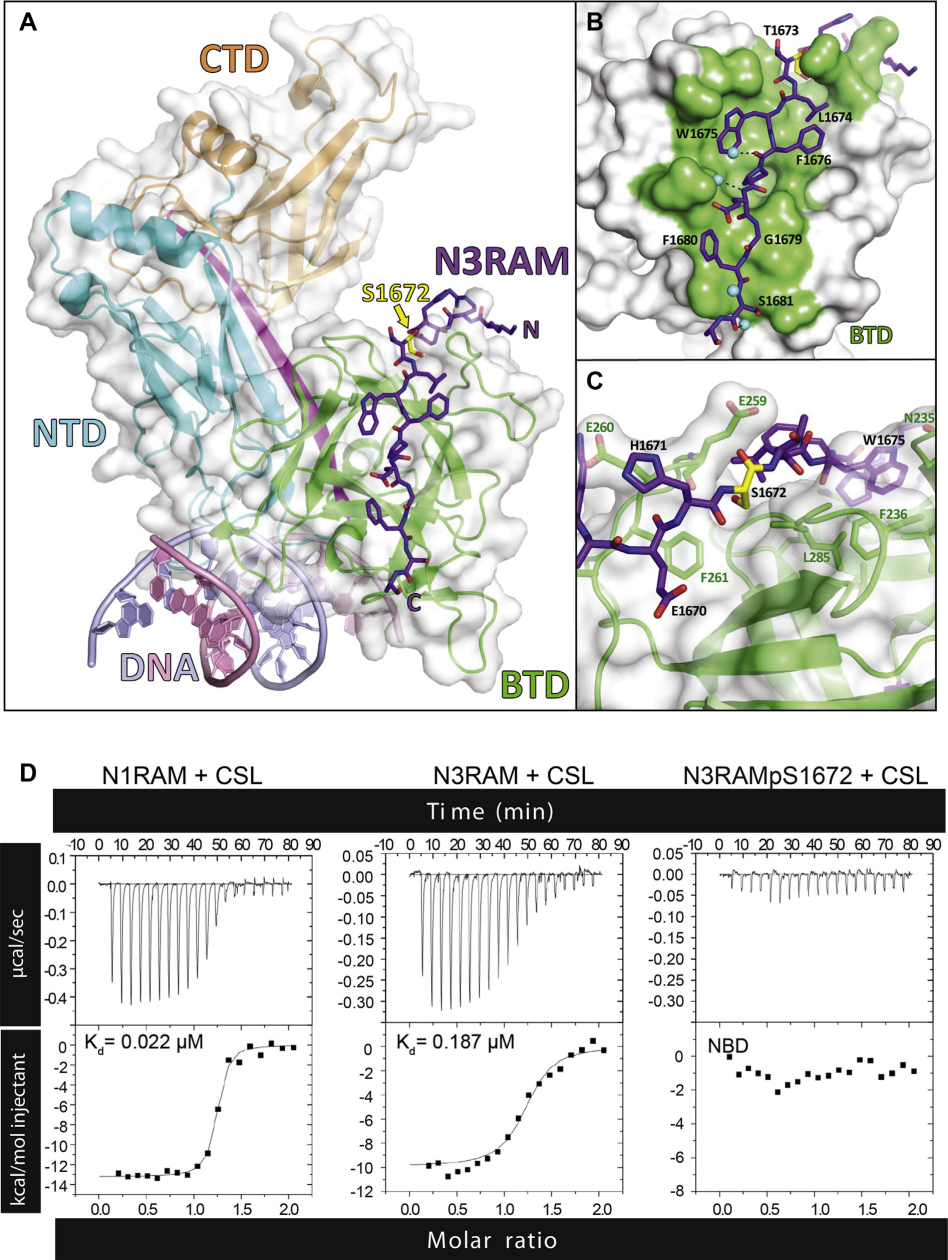
**Structure and binding properties of CSL and the NOTCH3 RAM (N3RAM) domain.***A,* CSL, shown with a transparent *white surface*, consists of three major domains: the N-terminal domain (NTD) in *cyan*, the beta-trefoil domain (BTD) in *green*, and the C-terminal domain (CTD) in *orange*. The N3RAM peptide corresponding to residues 1665 to 1682 is shown as a stick representation in *purple*. *B,* close-up view of the N3RAM bound to the BTD of CSL. Residues of CSL colored *green* directly contact RAM, as determined by the PISA server (15). *C,* close-up view of S1672 colored in *yellow*. The *dashed line* represents the hydrogen bond between the carbonyl group of the peptide backbone on E259 in CSL and the hydroxyl group of S1672 in N3RAM. Select CSL side chains that make up the hydrophobic pocket accommodating RAM S1672 are shown as stick representations in *green*. *D,* isothermal titration calorimetry experiments measuring CSL binding to RAM peptides. Representative thermograms from triplicate experiments of the binding of CSL to the NOTCH1 RAM residues 1754 to 1781 (*left*), nonphosphorylated N3RAM residues 1665 to 1682 (*middle*), and N3RAM residues 1665 to 1682 with a phosphorylation at S1672 (*right*). NBD signifies no binding detected.

To corroborate our predictive model, we crystallized mouse CSL (amino acid residues 53–474) in complex with the human N3RAM peptide (residues 1665–1682) (Fig. 4, *A*–*C*). These structural data also suggested that binding between N3RAM and CSL would be disrupted by phosphorylation at S1672. To confirm this, we performed isothermal titration calorimetry (ITC) on mouse CSL with human RAM peptides, using either N3RAM, phosphorylated N3RAMpS1672, or N1RAM (residues 1754–1781), which was used as a positive binding control. Binding between naïve CSL and N3RAM was weaker (*K*_*d*_ = 0.187 μM) as compared with binding between CSL and N1RAM (*K*_*d*_ = 0.022 μM) and, in line with our predictive model, no binding could be detected between CSL and the phosphorylated N3RAMpS1672 (Fig. 4*D* and Tables S2–S3). In line with this observation, we were unable to produce crystal structures of the phosphorylated human N3RAMpS1672 peptide in complex with CSL (data not shown). Taken together, the properties of the binding site are ideal for a small and hydrophobic residue, whereas phosphorylation at S1672 abolishes the binding capacity of N3RAM for CSL.

### Phosphorylation of Notch3 inhibits CSL-dependent transactivation

**Figure 5:**
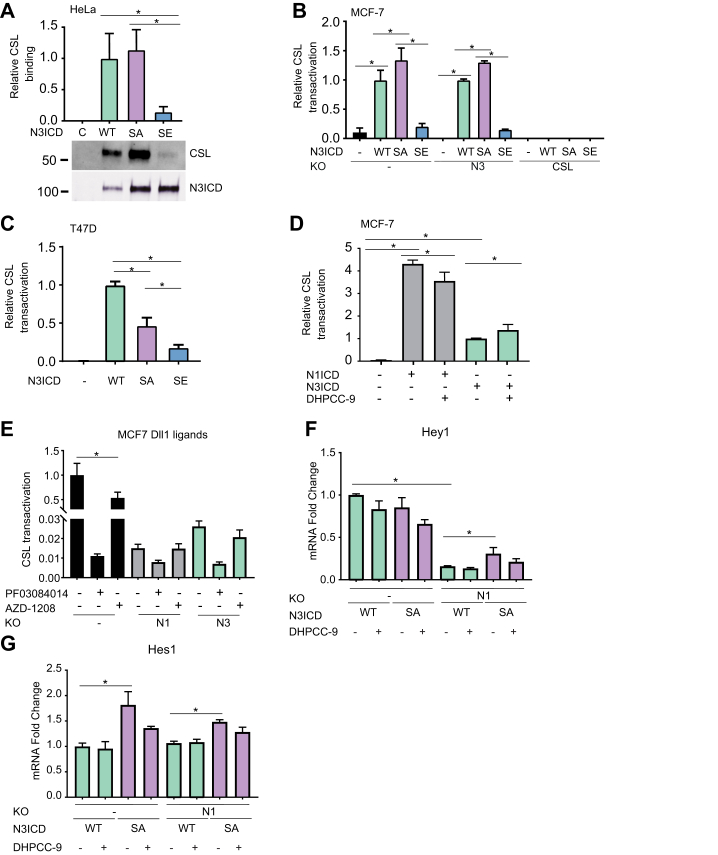
**Phosphorylation inhibits CSL-dependent transactivation by N3ICD but promotes tumor growth.***A,* empty vector (C), GFP-tagged WT, SA, or SE N3ICD and FLAG-tagged CSL were transiently overexpressed in HeLa cells. Interactions of N3ICD and CSL were analyzed by GFP-trap immunoprecipitation, followed by Western blotting with GFP (CSL) and FLAG (N3ICD) antibodies. Shown are average CSL binding values relative to WT N3ICD. *B* and *C,* CSL-dependent transactivation assays were performed in MCF-7 cells, their *NOTCH3* or *CSL*-deficient KO derivatives, or in T47D cells, which transiently overexpressed WT, phosphodeficient (SA), or phosphomimicking (SE) N3ICD. Luciferase activities were normalized according to β-galactosidase activities. Shown are averages relative to WT N3ICD from two independent experiments. *D,* CSL-dependent transactivation assays were also performed in MCF-7 cells transiently overexpressing N1ICD or N3ICD that were treated with DMSO or 10 μM DHPCC-9 for 16 h. *E,* endogenous NOTCH activity was measured in MCF-7 cells or their *NOTCH1* (N1) or *NOTCH3* (N3) KO derivatives plated on Delta-like 1 ligands (Dll1) and treated with DMSO, 10 μM PF03084014, or 10 μM AZD-1208. *F,* RT-quantitative PCR was performed to WT or N1KO MCF-7 cells transiently overexpressing WT or SA N3ICD. NOTCH target gene levels were normalized by *NOTCH3* levels after *UBC* subtraction. Shown are average values relative to DMSO-treated samples. All experiments had three parallel samples. ∗*p* < 0.05 was used as a limit for significant difference. Error bars represent standard deviations. N3ICD, Notch3 ICD; SA, serine to alanine; SE, serine to glutamic acid.

To assess the importance of N3ICD phosphorylation status on CSL binding in cells, we overexpressed FLAG-tagged CSL and GFP-tagged N3ICD proteins and analyzed their interactions by immunoprecipitation. In line with our ITC experiments, CSL coprecipitated with both the WT N3ICD and the phosphodeficient SA mutant, whereas hardly any coprecipitation was observed with the phosphomimicking SE mutant (Fig. 5*A*).

We next used CSL-dependent luciferase reporter assays to analyze the effect of N3ICD phosphorylation on transcriptional activity. The WT N3ICD, the phosphodeficient (SA) mutant, or the phosphomimicking (SE) mutant were transiently overexpressed in MCF-7 and T47D luminal A breast cancer cells as well as in the derivatives of MCF-7 cells, in which the *NOTCH3* or *CSL* genes had been knocked out by the CRISPR/Cas9 technique (Fig. S1). In MCF-7 cells, the phosphodeficient SA mutant of N3ICD activated the 12xCSL luciferase reporter even more efficiently than the WT N3ICD irrespective of the presence or the absence of endogenously expressed *NOTCH3* (Fig. 5*B*). By contrast, transactivation by the phosphomimicking SE mutant was severely compromised. As expected, no activity was detected in the KO cells lacking CSL protein. Similar results were obtained in T47D cells, except that WT N3ICD was more active than the SA mutant (Fig. 5*C*). This may be partly explained by the observation that there was less PIM protein expression as compared with MCF-7 cells (Fig. S5*B*) to regulate N3ICD activity in a phosphorylation-dependent fashion.

When comparing the activities of overexpressed N1ICD and N3ICD, we observed that both induced CSL-dependent transactivation but that N1ICD was approximately four times more active than N3ICD (Fig. 5*D*). However, PIM inhibition by the pan-PIM inhibitor DHPCC-9 reduced activity of N1ICD, but increased that of N3ICD, which was in line with the data on the overexpressed N3ICD phosphomutants (Fig. 5*B*). We then analyzed endogenous NOTCH activity in MCF-7 cells or their KO derivatives lacking either *NOTCH1* or *NOTCH3*. When these cell lines were cultured on plates coated with the DLL1 Notch ligand and treated with either DMSO, the Notch inhibitor PF03084014, or another pan-PIM inhibitor, AZD-1208, lack of either *NOTCH1* or *NOTCH3* abrogated NOTCH activity nearly to the same negligible level as inhibition of NICD cleavage by PF03084014 (Fig. 5*E*). As expected from our previous data (7), the PIM inhibitors reduced the NOTCH activity in parental MCF-7 cells but did not have any major effects in the KO cells.

To determine whether phosphorylation affects the expression of CSL-dependent Notch target genes, MCF-7 cells or their *NOTCH1*-deficient derivatives were transiently transfected with WT N3ICD or the SA mutant and treated with either DMSO or DHPCC-9 for 24 h. Real-time quantitative PCR revealed strong dependency of *HEY1* (hairy/enhancer-of-split related with YRPW motif protein 1) mRNA expression on NOTCH1, as demonstrated by its responsiveness to PIM inhibition as well as its remarkably reduced levels in the absence of *NOTCH1* (Fig. 5*F*). By contrast, *HES1* (hairy and enhancer of split 1) mRNA expression was more dependent on NOTCH3 activity, as there was no major difference between WT and N1KO MCF-7 cells. Interestingly, overexpression of the SA mutant upregulated both *HEY1* and *HES1* expression more efficiently than WT N3ICD. Taken together, these data demonstrate that PIM kinases support the CSL-dependent transcriptional activity of Notch1 but reduce the activity of Notch3.

### N3ICD phosphorylation promotes breast cancer tumorigenicity

**Figure 6:**
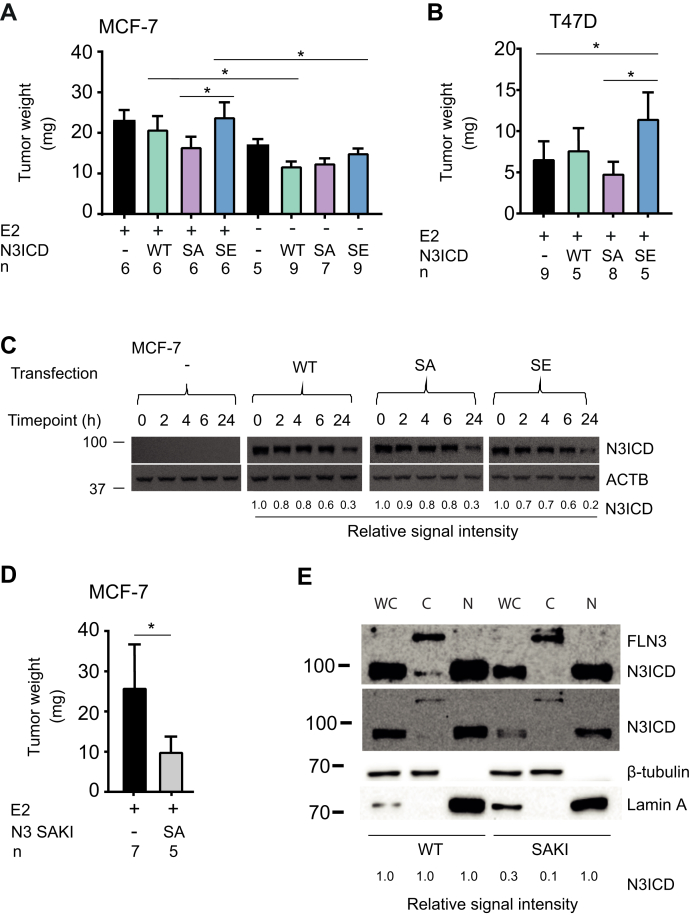
**Phosphorylation promotes Notch3 ICD (N3ICD) tumorigenicity on chorioallantoic membrane (CAM).***A* and *B,* MCF-7 or T47D cells were transiently transfected to overexpress WT, SA, or SE N3ICD, then grown for 5 days on the CAMs of chick embryos in the presence (+) or absence (−) of 100 μM estradiol before weighing tumors. Egg numbers (n) are shown under the graph bars. ∗*p* < 0.05 was used as a limit for significant difference. Error bars represent standard deviations. *C,* MCF-7 cells transiently overexpressing FLAG-tagged WT or mutant N3ICD were treated with 15 μg/ml cycloheximide, after which cells were lysed at the defined time points. Levels of N3ICDs in these lysates were then determined by Western blotting. Actin beta (ACTB) staining was used as a loading control. *D,* parental MCF-7 cells or their knock-in (SAKI) derivatives endogenously expressing the phosphodeficient (SA) mutant of NOTCH3 were grown for 5 days on CAM in the presence (+) of 100 μM estradiol before weighing tumors. *E,* nuclear fractionation was performed to WT or SAKI MCF-7 cells, after which full-length NOTCH3 (FLN3) or N3ICD was detected from the whole cell lysates (WC), cytoplasm (C), or nucleus (N). Fractionation and protein loading were controlled for by ß-tubulin and lamin A levels. Shown is a representative immunoblotting example from two similar experiments. SA, serine to alanine; SE, serine to glutamic acid.

To assess the physiological role of Notch3 phosphorylation *in vivo*, WT or phosphomutant forms of N3ICD were transiently overexpressed in MCF-7 or T47D cells, followed by transplantation of the cells onto the chorioallantoic membranes (CAMs) of fertilized chicken eggs, and the growth of the xenografted cells was followed for 5 days. The CAM model was chosen for this purpose as it offers a fast and easy *in vivo* system to analyze the tumorigenic potential of transiently transfected cells. As the growth of MCF-7 and T47D cells is estrogen-dependent, larger tumors were obtained when the xenografts were treated with 100 μM estradiol (E2; Fig. 6, *A* and *B*). In the presence of E2, overexpression of WT N3ICD or the phosphomimicking SE mutant supported tumor growth, whereas the phosphodeficient SA mutant abrogated it to the same extent as lack of E2. By contrast, in the absence of E2, all N3ICD variants reduced tumor growth.

As additional controls, cycloheximide pulse-chase experiments followed by Western blotting were conducted to demonstrate that the expression levels or intrinsic stability of the FLAG-tagged Notch3 proteins were not influenced by PIM-mediated phosphorylation (Fig. 6*C*).

To further confirm the relevance of PIM-mediated phosphorylation, we used the CRISPR/Cas9 technique to prepare MCF-7–based knock-in (KI) mutant cells, where the PIM-targeted serine 1672 of the endogenously expressed human NOTCH3 was mutated to alanine to create a cell line named SAKI (serine to alanine KI mutant) (Fig. S4). After validation of one positive cell clone with the desired mutation plus an additional conservative missense mutation (Fig. S5, *A* and *C*), we xenografted WT and SAKI cells on CAM and assessed tumor growth. The SAKI cells expressing the phosphodeficient SA mutant generated smaller tumors in response to E2 than parental MCF-7 cells (Fig. 6*D*), suggesting that phosphorylation of N3ICD supports its oncogenic activity.

As an additional control experiment, we fractionated WT and SAKI cells and analyzed by Western blotting the endogenous NOTCH3 protein levels. The levels of the full-length NOTCH3 protein were similar in both cell lines. Unexpectedly, the cytoplasmic levels of N3ICD were decreased in the SAKI cells as compared with WT cells, whereas the nuclear N3ICD levels remained relatively similar in both cell lines (Fig. 6*E*). These data suggest that in addition to affecting the nuclear transactivation activity of N3ICD, phosphorylation may also play a role in its stabilization in the cytoplasm.

## Discussion

**Figure 7:**
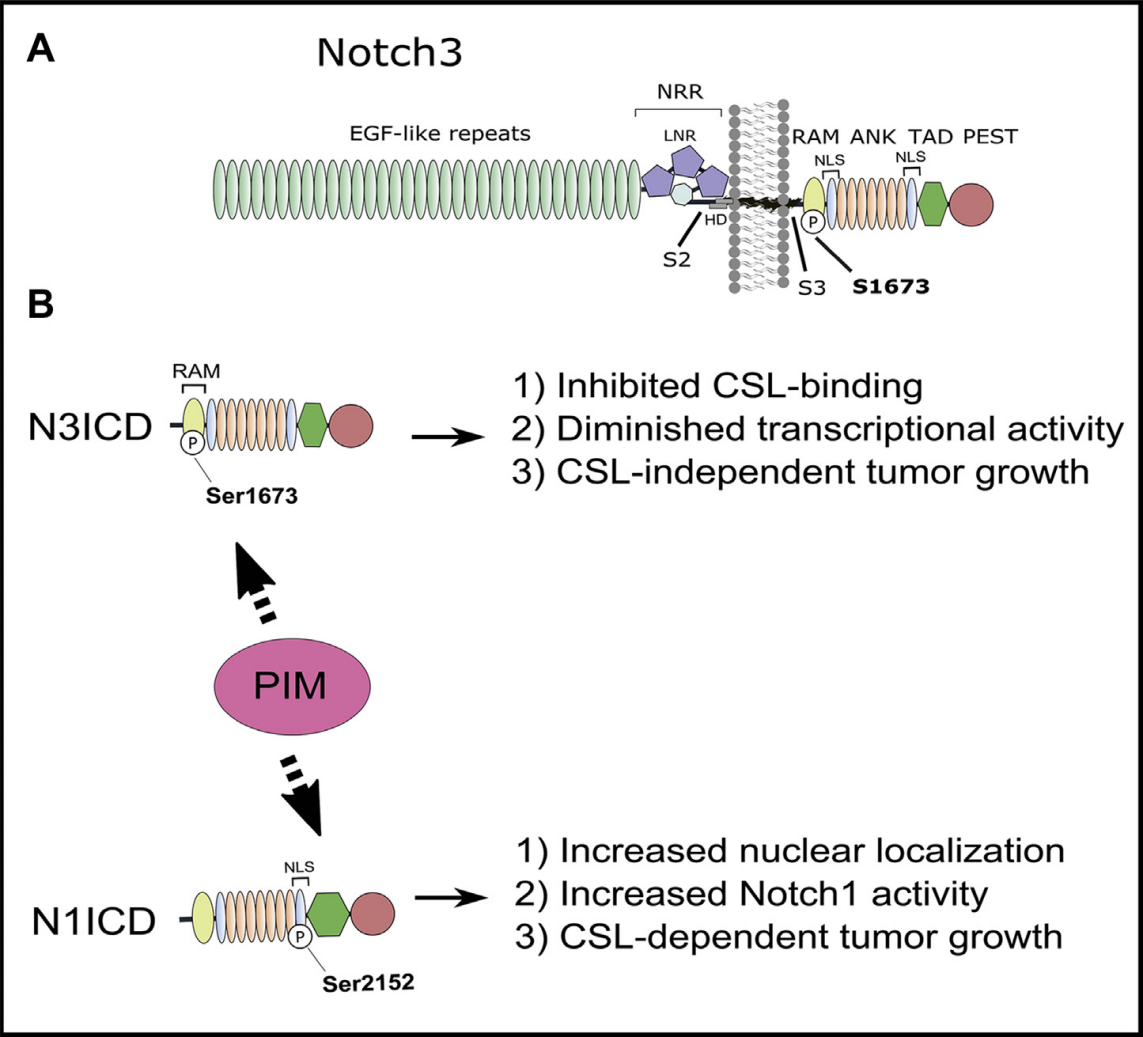
**Phosphorylation of Notch1 ICD (N1ICD) and Notch3 ICD (N3ICD) by proviral integration site for Moloney murine leukemia virus (PIM) kinases occurs in different domains and results in differential Notch signaling outputs.***A,* a schematic model of mouse Notch3 with the PIM target site at S1673. *B,* comparison of mouse N1ICD and N3ICD phosphorylation sites and their outcomes. PIM-mediated phosphorylation inhibits CSL-dependent activity of N3ICD but promotes that of N1ICD (7). In both cases, phosphorylation leads to increased tumor growth, albeit *via* different mechanisms. ANK, ankyrin repeat domain; CSL, C promoter–binding factor 1, Suppressor of Hairless, Lag-1; EGF, epidermal growth factor; HD, heterodimerization domain; LNR, Lin12-Notch repeat; NECD, Notch extracellular domain; NLS, nuclear localization sequence; NRR, negative regulatory region; PEST, proline-, glutamic acid-, serine-, and threonine-rich domain; RAM, RBPJ-associated molecule domain; S2, ADAM family metalloprotease cleavage site; S3, γ-secretase cleavage site; TAD, transcription activation domain; TM, transmembrane domain.

We have previously shown that PIM kinases phosphorylate mouse Notch1 on serine 2152 (corresponding to human NOTCH1 S2162) within the second nuclear localization sequence, promoting its nuclear translocation and CSL-dependent transcriptional activity (7). Here, we demonstrate that PIM kinases phosphorylate Notch3 at a distinct site (serine 1673; corresponding to human NOTCH3 S1672) within the RAM domain, which is essential for binding to CSL (17, 18, 19) (Fig. 7*A*). Phosphorylated N3ICD cannot bind CSL and therefore remains transcriptionally inactive but nonetheless promotes cell survival and tumor growth under estrogenic conditions (Fig. 7*B*). Conversely, the stronger transactivation potential observed with the SA mutant as compared with WT N3ICD in MCF-7 cells suggests that nonphosphorylated N3ICD is the driver of CSL-dependent canonical Notch3 signaling, whereas PIM kinases act as a brake to inhibit Notch3 transcriptional output in the nucleus.

The high structural and sequence similarity between NOTCH1 and NOTCH3 allowed us to model the human NOTCH3 transcriptional complex superimposed on the NOTCH1 complex (16). Based on mutagenesis of the RAM domain, binding to CSL is dependent on the XWXP motif (where X is a hydrophobic residue), the N-terminal basic region, the His–Gly motif, and the C-terminal Gly-Phe dipeptide (19). All these regions are conserved in N3ICD, except for the His–Gly motif, where the glycine residue is replaced by the PIM-targeted serine residue. Upon phosphorylation of N3ICD, its His-Ser motif, size, and charge restrictions are likely to cause a conformational change and a displacement of the RAM peptide. This was confirmed by crystallization of human N3RAM peptides in complex with mouse CSL, as the phosphorylated N3RAMpS1672 peptide was unable to bind to CSL. Taken together, our data indicate that phosphorylated N3ICD is unable to form a stable nuclear complex with CSL, resulting in a marked reduction in CSL-dependent transactivation.

While *PIM* upregulation has been associated with malignant breast cancer (20, 21, 22), the role of *NOTCH3* has remained controversial. Dysregulated *NOTCH3* expression has often been linked to a more aggressive disease (23), and in triple-negative breast cancer reported as an oncogene (24, 25), while in luminal breast cancer, *NOTCH3* upregulation has been associated with increased relapse-free survival (26, 27). Here, we show that high levels of *PIM* mRNA are often connected to better survival, but that in the special subgroup of grade 3 estrogen-positive breast cancer, upregulated expression of both *PIM1* and *NOTCH3* mRNAs predicts poor survival. Furthermore, in clinical data from luminal A subtype of breast cancer, *PIM1* and *NOTCH3* mRNA levels correlate positively. All these data support our observations that in luminal A breast cancer cells, NOTCH3 can act as an oncogenic protein when it is phosphorylated at serine 1672, whereas the nonphosphorylated form of N3ICD remains tumor-suppressive.

Thus far, reports on the tumor-suppressive role of NOTCH3 have mainly focused on the canonical CSL-dependent signaling (26, 28). According to our data, the phosphorylated N3ICD with reduced CSL-dependent transcriptional activity enhances estrogen-driven tumorigenic growth of breast cancer cells. Thus, the NOTCH3 output in luminal A breast cancer may be determined by the activity of kinases such as PIM that phosphorylate N3ICD at serine 1672. Additional studies are required to elucidate the physiologically relevant stoichiometry of PIM-dependent phosphorylation and its significance in biological settings. However, the concept of kinases acting as switches in the Notch pathway holds promise of yielding novel therapeutic concepts in the future, including the utilization of intracellular antibodies or intrabodies to block paralog-specific Notch outputs (6).

In conclusion, we have revealed evolutionarily divergent PIM target sites on Notch3 as compared with Notch1 and shown that phosphorylation has differential effects on their transcriptional activity, even though it promotes tumorigenesis in both cases.

## Experimental procedures

### Clinical dataset analyses

For database analyses, datasets were exported from cBioPortal and kmplot.com (11, 12) and further processed by R, version 3.6.3 (29) by RStudio, version 1.2.5033 (RStudio, Inc). Graphical data were exported as svg files for figure preparation.

Gene expression data (mRNA expression z-scores relative to all samples, loq RNA Seq V2 RSEM) and clinical data (Oncoprint with all clinical tracks) were obtained from cBioPortal database from Cancer Genomics, Breast Invasive Carcinoma (TCGA, PanCancer Atlas) dataset. Expression and clinical data were merged and analyzed in R. The packages plyr (revalue function), ggpubr (ggdensity function for normality testing), and Hmisc (rcorr function for Pearson's correlation coefficiency) were used.

Correlation of gene expression levels to overall survival was analyzed by kmplot.com Kaplan–Meier Plotter mRNA gene chip, Breast Cancer. Probe Id 209193_at was selected for PIM1 and 203237_s_at for NOTCH3. Patients were split by Auto select best cutoff, and analyses were performed to all data or restricted to subgroups. Data were exported as txt, and plots were prepared in R using packages survival and survminer (functions survfit and ggsurvplot).

### *In vitro* kinase assays and *in silico* analyses

The glutathione *S*-transferase fusion protein production and radioactive *in vitro* kinase assays were carried out as previously described (30). Western blotting with PAS antibody (RXXS∗/T∗, 110B7E, #9614; Cell Signaling Technology, Inc) and Ponceau S staining (Sigma–Aldrich) was used for nonradioactive detection of phosphorylated and total proteins according to the manufacturer's protocols. Signal intensities were analyzed by the ChemiDoc MP Imaging System with Image Lab software, version 4.0 (Bio-Rad Laboratories, Inc). For *in silico* analysis, protein sequences were obtained from Uniprot Swiss-Prot Universal Protein Resource Knowledgebase (31). Potential PIM target sites were searched for according to the published consensus sequences (13, 32, 33) and by using the Human Protein Reference Database, PhosphoMotif Finder (34).

### DNA constructs and mutagenesis

Expression vectors pcDNA3.1/V5-His-C, pGEX-6P-1, and pTagRFP-N for WT and kinase-deficient human PIM kinases as well as pGEX-6P-3 and p3xFLAG-CMV-7.0 for mouse N3ICD have been previously described (7, 35). N3ICD was cleaved from p3xFLAG-CMV by HindIII and BamHI and ligated into pEGFP-C1 (Clontech). Ultra Pfu DNA polymerase was used according to the manufacturer's protocol (Stratagene) for site-directed mutagenesis of N3ICD. The mutagenesis primers are described in Table S4. Mouse CSL amino acid residues 53 to 474 corresponding to the conserved and structurally ordered core domain were cloned into the pSMT3 expression vector (36).

### Cell lines and treatments

Human MCF-7 and T47D breast cancer cells and HeLa cervical cancer cells (American Type Culture Collection) were cultured and transfected by electroporation or Fugene HD as previously described (7, 37). To inhibit the catalytic activity of PIM kinases, cells were treated with the small molecule pan-PIM inhibitors DHPCC-9 (38, 39) or AZD-1208 (AstraZeneca), whereas Notch activity was blocked by the γ-secretase inhibitor PF-03084014 (MedChemExpress). To determine the stability of target proteins, cells were treated with 15 μg/ml of the protein synthesis inhibitor cycloheximide over a period of 24 h. Cells were lysed at the given time points, and these lysates were processed for Western blotting. The xenografted cells grown on the CAMs were treated with estradiol (E2, E8875; Sigma–Aldrich), as previously described (7).

The stable MCF-7–based KO and KI cell lines were created by the CRISPR-/Cas9-based genome editing technique, as described in Tables S5–S8.

### Immunoprecipitation

Cells were lysed in 50 mM Tris–HCl, pH 7.5 buffer containing 10% glycerol, 100 mM NaCl, 1 mM EDTA, 1% NP-40, Mini-EDTA Free Protease inhibitors (11836170001; Roche), 50 mM NaF, 0.5 mM natriumpyrophosphate, and 1 mM Na_3_VO_4_. Protein concentrations were determined using the Bio-Rad Protein Assay Dye Reagent according to manufacturer's protocol (Bio-Rad Laboratories, Inc). For immunoprecipitation of FLAG-tagged N3ICD, 500 μg of protein was combined with 50 μl of anti-FLAG M2 affinity agarose gel (A2220; Sigma–Aldrich) in 1 ml of lysis buffer. After 1 h rotation at +4 °C, the agarose gel was washed four times with the lysis buffer. For immunoprecipitation of His-tagged PIM1, 500 μg of protein was combined with 50 μl of HisLink Protein Purification Resin (Promega) in 500 μl of lysis buffer supplemented with 10 mM imidazole (104716; Merck). Samples were incubated in rotation at +4 °C for 30 min, after which resin was washed four times with 20 mM imidazole in 10 mM Tris–HCl, pH 7.5. His-linked protein elution was performed in rotation at +4 °C for 30 min in 10 mM Tris–HCl, pH 7.5 buffer with 300 mM imidazole, and 250 mM NaCl. For immunoprecipitation of GFP-tagged proteins, GFP-trap IP system (ChromoTek, Inc) was utilized according to manufacturer's protocol. Samples were prepared for Western blotting by addition of preheated 2× Laemmli Sample Buffer, vortexing and heating for 5 min at +95 °C.

### Protein expression and purification

Mouse CSL amino acid residues 53 to 474 corresponding to the conserved and structurally ordered core domain were cloned into the pSMT3 expression vector. BL21(DE3) cells transformed with pSMT3-mCSL were grown in LB to an absorbance of 1.5 followed by IPTG induction. Cell pellets were resuspended in lysis buffer, sonicated, and centrifuged at 15,000*g* for 40 min. About 60% w/v ammonium sulfate was added to the supernatant to precipitate the protein and then centrifuged at 11,000*g* for 45 min. The protein pellet was resuspended in binding buffer (20 mM Tris pH 8, 50 mM imidazole, 0.5 M NaCl, and 0.1% Triton) and incubated overnight with nickel affinity resin. His-tagged protein was eluted from the nickel affinity resin and then cut with ubiquitin-like protease to remove the SMT3 tag. A sulphopropyl ion exchange column was used to separate the cut SMT3 from the CSL protein. CSL was then sized on an S200 16/60 sizing column into buffer containing 20 mM Tris pH 8, 0.5 M NaCl, 1 mM EDTA, 1% ethylene glycol, and 0.1 mM Tris(2-carboxyethyl)phosphine. CSL was then concentrated and flash frozen in liquid nitrogen and stored at −80 ˚C until further use.

### Peptide synthesis

Human NOTCH1 residues 1754 to 1781 (VLLSRKRRRQHGQLWFPEGFKVSEASKK) and NOTCH3 residues 1665 to 1682 (ARRKREHSTLWFPEGFKV, phosphorylated or nonphosphorylated at S1672) corresponding to the RAM domains were synthesized with 95% purity by Peptide2.0 and further purified by vacuum centrifugation before ITC.

### Oligonucleotide preparation

The following 15-mer oligonucleotide sequences were ordered from Eurofins Scientific (Luxembourg): 5’-TTACCGTGGGAAAGA-3’ and the reverse complementary sequence 5’-AATCTTTCCCACGGT-3’ showing the CSL-binding site underlined. Single-stranded oligonucleotides were further purified on a Resource Q ion exchange column and then buffer exchanged into a buffer containing 10 mM Tris pH 8.0, 500 mM NaCl, and 1 mM MgCl_2_. Single-stranded oligonucleotides were added together in equal molar amounts and boiled for 10 min and then slowly cooled to room temperature to allow for proper annealing.

### Crystallization and data collection

Crystallization experiments were set up under paraffin oil with CSL at 150 μM, N3RAM at 170 μM, and 15-mer DNA at 165 μM. The mother liquor solution contained 0.2 M ammonium fluoride and 14% PEG 3350. Crystals were slowly transferred into the mother liquor supplemented with 20% xylitol for cryoprotection before freezing in liquid nitrogen. Diffraction data were collected at the Advanced Photon Source, beamline 24-ID-C-NE. The CSL/N3RAM/DNA crystals diffracted to 2.4 Å, belonged to the space group P 2_1_2_1_2_1_, and had unit cell dimensions of a = 66.45 Å, b = 97.66 Å, and c = 104.62 Å.

### Structure determination and model building

Diffraction data were indexed, integrated, and scaled in iMosflm (40), and molecular replacement was performed in Phaser (41) using the structure of CSL bound to the *HES1* DNA site (Protein Data Bank [PDB]: 3IAG) combined with the *Caenorhabditis elegans* structure of the Lin-12 RAM peptide (PDB: 3BRF) as the search model. Refinement was initially performed in Phenix (42) along with manual model building in Coot (43). The model was further refined using BUSTER (44) and validated with MolProbity (45). The final structure of CSL/N3RAM/DNA was refined to an *R*_work_ = 19.88% and *R*_free_ = 23.34%. All structure figures were generated in PyMol (Schrödinger, Inc), and protein interfaces were analyzed with the PISA server (Macromolecular Structure Database, European Bioinformatics Institute) (46).

### ITC

CSL and NOTCH RAM peptides were dialyzed overnight in 50 mM sodium phosphate and 150 mM sodium chloride buffer. Experiments were conducted using the VP-ITC MicroCalorimeter manufactured by MicroCal. All experiments were performed at 20 °C with CSL in the cell at 10 μM and RAM peptides in the syringe at 100 μM. Each experiment was performed in triplicate with 20 injections, 14 μl per injection. Heat of dilution experiments were performed by injecting syringe samples into a cell containing only buffer, and all analyses were performed with the heat of dilution subtracted before fitting.

### Immunoblotting, immunofluorescence, and fluorescence microscopy

Western blotting was performed as previously described (37), while details for the antibody dilutions, enhanced chemiluminescence, and imaging are described in Table S9. Transiently overexpressed RFP- or GFP-tagged proteins were used for analysis of localization and interactions by confocal microscopy or fluorescence-lifetime imaging, whereas proximity ligation assay was used to show protein interactions utilizing antibodies targeting endogenous NOTCH3 (A-6 sc-515825; Santa Cruz Biotechnology and ab23426; Abcam) and/or PIM1 (H00005292-M16, clone 6A2; Novus Biologicals), as previously described (6). Image and correlation analyses were performed by ImageJ (1.48s; Fiji, Wayne Rasband, National Institutes of Health).

### Transactivation assays

Notch activity was measured from cells transiently transfected with the 12xCSL-luciferase (RBPJ) (47) and the β-galactosidase reporter constructs, using the Luciferase Reporter Gene Detection Kit (LUC1-1KT; Sigma–Aldrich) or VivoGlo Luciferin (Promega) according to the manufacturers' instructions. Normalization by β-galactosidase activity and DLL1 coating was carried out as previously described (37, 48).

### RT-quantitative PCR

RNA was extracted with the NucleoSpin RNA kit (Macherey-Nagel) according to the manufacturer's instructions. Complementary DNA samples were prepared using the SensiFAST cDNA Synthesis Kit (Meridian Bioscience) according to the manufacturer's instructions from equal amounts of RNA. The reaction mixtures for quantitative PCR were prepared with 5× HOT FIREPol EvaGreen qPCR Mix Plus (Solis BioDyne) according to the manufacturer's instructions (Sigma–Aldrich). RNA and H_2_O controls were included to ensure that RNA preparations and PCR mixtures were not contaminated. The primer sequences for detection of *NOTCH1*, *NOTCH3, HEY1, HES1*, and *UBC* (a house-keeping control) mRNAs have been described in Table S10.

### Animal models for human breast cancer

For chicken egg experiments, cells were inoculated onto chick embryo CAMs, grown, and analyzed as previously described (7). Mouse experiments were authorized by the National Animal Experiment Board in accordance with The Finnish Act on Animal Experimentation (Animal license number: 10438/04.10.07/2016). Athymic Nude-*Foxn1nu* female mice (Envigo) were housed under controlled conditions and supplemented with estradiol pellets as previously described (37). Two weeks after pellet implantation, mice were randomly divided into three groups and under anesthesia injected into their bilateral upper inguinal mammary glands with 2.5 × 10^6^ MCF7 cells or their derivatives mixed 1:1 with Matrigel (BD Biosciences). At the time of xenotransplantation, mice were 7 weeks old. Tumor measurements and animal sacrifice were performed as previously described (37).

### Statistical analysis and figure preparation

Bar graphs and scatter plots were produced by R, version 3.6.3. (The R Foundation), Microsoft Excel 2016 (Microsoft Corporation), or GraphPad Prism 4.00 (GraphPad Software), and results were analyzed by Student's *t* test or ANOVA. Pearson's correlation coefficiency was defined as *very strong* (*R*^2^ ≤ 0.800), *strong* (*R*^2^ = 0.600–0.799), *moderate* (*R*^2^ = 0.400–0.599), or weak (*R*^2^ = 0.2–0.399). Figures were prepared by CorelDRAW Graphics Suite 2020 (Corel Corporation) or Adobe Illustrator CS5 15.0.0 (Adobe).

## Data availability

The completed refined structure of CSL/N3RAM/DNA was deposited into the PDB with accession code 6WQU. Detailed code for R is available in Github (https://github.com/nmsantio/Gene-expression-analysis).

## Supporting information

This article contains supporting information.

## Conflict of interest

U. L. holds research grants from Merck KGaA, no personal remuneration. All other authors declare that they have no conflicts of interest with the contents of this article.
