# Supplementary material for: PIM-induced phosphorylation of Notch3 promotes breast cancer tumorigenicity in a CSL-independent fashion
Source: J Biol Chem. 2021 Mar 26;296:100593. doi: 10.1016/j.jbc.2021.100593 (PMC8100066; doi:10.1016/j.jbc.2021.100593)
Supplement: Supplementary material [file mmc1.pdf]

SUPPORTING INFORMATION

PIM-induced phosphorylation of Notch3 promotes breast cancer tumorigenicity in a CSL-independent fashion

Sebastian K.J. Landor, Niina M. Santio, William B. Eccleshall, Valeriy M. Paramonov, Ellen K. Gagliani, Daniel Hall, Shao-Bo Jin, Käthe M. Dahlström, Tiina A. Salminen, Adolfo Rivero-Müller, Urban Lendahl, Rhett A. Kovall, Päivi J. Koskinen, Cecilia Sahlgren

CONTENTS

Supplementary Experimental procedure 1. Schematic and structural protein modeling

Supplementary Experimental procedure 2. Establishment of stable CRISPR knock-out and knock-in cell lines

Supplementary Table S1. *PIM1* and *NOTCH3* connection to overall survival in breast cancer.

Supplementary Table S2. X-ray Data collection and refinement statistics.

Supplementary Table S3. Calorimetric binding data for NOTCH RAM domain peptides to CSL.

Supplementary Table S4. Primers for site-directed mutagenesis of plasmid DNA.

Supplementary Table S5. Guide RNA sequences for CRISPR/Cas9-mediated knock-out mutagenesis.

Supplementary Table S6. Design of the *NOTCH3 S1672A* targeting plasmid.

Supplementary Table S7. Assembly of the template plasmid for knock-in mutagenesis.

Supplementary Table S8. Primers utilized in the knock-in mutagenesis.

Supplementary Table S9. Antibody dilutions and procedures used for Western blotting**.**

Supplementary Table S10. Primers used for qPCR.

Supplementary Figure S1. Verification of *NOTCH* and *CSL* knock-out cell lines.

Supplementary Figure S2. *PIM1* and *NOTCH3* expression correlates in breast cancer stages I-III and Luminal A subtype.

Supplementary Figure S3. Model predicting the binding conformations of non-phosphorylated and phosphorylated NOTCH3 RAM to CSL.

Supplementary Figure S4. Validation of the template plasmid assembly for endogenous NOTCH3 S1672A knock-in mutagenesis.

Supplementary Figure S5. Characterization of the NOTCH3 E1678D mutation and cellular PIM expression levels.

Schematic and structural protein modeling

Schematic modelling of human NOTCH3 was performed by the Inkscape 0.92.3 vector graphics software (inkscape.org). The crystal structure of the human NOTCH1 transcription complex (PDB 3V79) [1] was used as a template for modeling of the corresponding complex of human NOTCH3 (UniProtKB Q9UM47). MALIGN in the BODIL modeling environment [2] was used to align NOTCH1 (UniProtKB P46531) and NOTCH3 ANK (ankyrin) and RAM (RBPJ-associated molecule) domains. Based on the alignments and the template, the structures of NOTCH3 ANK and RAM domains were modeled with the Homodge program in Bodil [2], while the coordinates for DNA, MAML and CSL were copied directly from the template (PDB 3V79) [1]. The resulting structural model of the NOTCH3 transcription complex was minimized in Maestro (Version 11.4.011, Schrödinger, Inc., New York, NY, USA) with force field OPLS3, water as solvent and maximum iterations 2500. To analyze the effect of S1672 phosphorylation, a phosphoserine from the crystal structure of human PIM1 kinase (PDB 5N5M) [3] was superimposed on top of S1672 in the 3D model of the minimized NOTCH3 transcription complex and the coordinates for the phosphate group were copied and linked to S1672. The NOTCH3 transcription complex with phosphorylated S1672 was subsequently minimized as previously described and compared to the non-phosphorylated complex by superimposition of the CSL subunits with the VERTAA software [4] in Bodil. The APBS tool in PyMOL (Version 2.1.0, Schrödinger, LLC.) was used to calculate the electrostatic surface potential with PyMOL generated PQR, hydrogens and termini. Pictures were prepared with PyMOL (Version 2.1.0, Schrödinger, LLC.).

Establishment of stable CRISPR knock-out and knock-in cell lines

The stable MCF-7-based knock-out cell lines were created by the CRISPR/Cas9-based genome editing technique. The guide RNAs (gRNAs) are described in **Table S5**. For *NOTCH1* knock-out, the gRNAs were selected by the CRISPOR software [5] and the gRNAs were acquired as gBlocks gene fragments (Integrated DNA Technologies, Coralville, Iowa, USA). The gRNAs were ligated for *NOTCH1* knock-out to the BbsI-digested pSpCas9(BB)-2A-GFP (PX458) [6] to simultaneously express two single gRNAs. After transformation, the bacterial colonies were screened by PCR and Sanger sequencing using forward 5'- ACGATACAAGGCTGTTAGAGAGAT -3' and reverse 5'- ACGCGCTAAAAACGGACTAGC -3' primers. For generation of *NOTCH1* stable CRISPR knock-out cell line, MCF-7 cells were transfected with Lipofectamine (4:1 to DNA; Thermo Fisher Scientific) according to manufacturer's protocol. PCR amplification was used to exclude bi-allelic loss of large gene fragments, resulting in similarly sized bands for the genomic DNA of wild-type and knock-out cells. All mutant clones were verified by Sanger sequencing of the genomic DNA using forward 5' - ATGTTGACGCCCCTTTGGT -3' and reverse 5'- TCTGCCCTCGACAAAGCAAC - 3' primers.

The gRNA for *CSL* knock-out as well as the procedure for *CSL* and *NOTCH3* knock-outs have been described earlier for a different cell line [7]. The knock-in *NOTCH3* (NCBI gene ID: 4854) S1672A mutation (AGC>GCT) was introduced by the CRISPR/Cas9 technology. The targeting plasmid encoded *S. pyogenes* Cas9 nuclease, while three guide RNAs (gRNAs) were selected by the CRISPOR software [5] for *NOTCH3* exons 27-28 (**Table S6**). The template plasmid was constructed from five DNA fragments within a single Gibson assembly reaction [8]. It carried parts of mutated *NOTCH3* coding DNA sequence (the transgene) and recoded sites for gRNAs to prevent Cas9 recruitment to the integrated transgene. Long homology arms were added to facilitate homology-directed repair **(Table S7),** while loxP-flanked accessory parts were added to aid in clonal isolation and screening by fluorescence-associated cell sorting (FACS). The template was validated by PCR and Sanger sequencing**.** The primers used are described in **Table S8.**

For production of the knock-in cell line, MCF-7 cells were co-transfected with the targeting and template plasmids by FuGENE® HD 1:1 to DNA (Promega). Selection started 72 hours later by 75 μg/ml of hygromycin B (#10687-010, Invitrogen). After 13 days of selection, cells were sorted by FACS according to mCardinal signal (FACSAria II cell sorter with 100 μm nozzle; Becton Dickinson, USA)

into polyclonal subpopulations. After expansion, the subpopulation carrying the insertion was further used for single cell FACS. The emerged monoclonal populations were further screened and expanded. Then, cells were seeded into a six-well plate (600 000 cells/ well) and after 24 hours, co-transfected by FuGENE® HD 3:1 to DNA (5 µg/ well) with plasmids encoding Cre recombinase and mCardinal fluorescent protein (#51311, Addgene). After 72 hours, cells were sorted by FACS and seeded one/well into 96-well plates. The expanded single cell-derived populations were screened to identify subclones, where the accessory parts were excised by Cre and the mutation S1672A was successfully introduced. Based on Sanger sequencing, only one correct subclone was identified, but it also contained an additional A to C substitution leading to a conservative missense mutation of E1678 to D. For functional characterization of this mutation, the equivalent amino acid E1679 in mouse N3ICD was replaced with D by site-directed mutagenesis, which was performed according to the REPLACR approach [9] to Notch3-ICD-pEGFP-C1, using the high-fidelity KOD Xtreme polymerase (Sigma Aldrich, St.Louis, MI, USA) with the following mutagenesis primers: F: 5' –GTTCCCTGAC GGTTTTGCATTACAC -3' and R: 5'- CAAAACCGTCAGGGAACCAGAGG -3'.

References

1. Choi SH, Wales TE, Nam Y, O'Donovan DJ, Sliz P, Engen JR *et al.* Conformational locking upon cooperative assembly of Notch transcription complexes. *Structure* 2012; **20**: 340–349.
2. Lehtonen JV, Still DJ, Rantanen VV, Ekholm J, Björklund D, Iftikhar Z, *et al.* BODIL: a molecular modeling environment for structure-function analysis and drug design. *J Comput Aided Mol Des* 2004; **18**: 401–419.
3. Siefker C, Heine A, Taylor C, Kolb P, Hardes K, Steinmetzer A *et al.* A crystallographic fragment study with human Pim-1 kinase. To be published. DOI: 10.2210/pdb5n5m/pdb
4. Johnson MS, Lehtonen JV. Comparison of protein three-dimensional structures. In: Higgins D, Taylor W (eds). *Bioinformatics: Sequence, structure and databanks.* Oxford University Press: Oxford, UK, 2000, pp. 15.
5. Haeussler M, Schönig K, Eckert H, Eschstruth A, Mianné J, Renaud JB *et al*. Evaluation of off-target and on-target scoring algorithms and integration into the guide RNA selection tool CRISPOR. *Genome Biol* 2016; **17:** 1–12.
6. Ran FA, Hsu PD, Wright J, Agarwala V, Scott DA, Zhang F. Genome engineering using the CRISPR-Cas9 system. *Nat Protoc* 2013; **8**: 2281-308.
7. Braune EB, Tsoi YL, Phoon YP, Landor S, Silva Cascales H *et al.* Loss of CSL unlocks a hypoxic response and enhanced tumor growth potential in breast cancer cells. *Stem Cell Reports* 2016; **6**: 643-651.
8. Gibson DG, Young L, Chuang R-Y, Venter JC, Hutchison CA, Smith HO. Enzymatic assembly of DNA molecules up to several hundred kilobases. *Nat Methods* 2009; **6:** 343–5.
9. Trehan A, Kiełbus M, Czapinski J, Stepulak A, Huhtaniemi I, Rivero-Müller A. REPLACR-mutagenesis, a one-step method for site-directed mutagenesis by recombineering. *Sci Rep.* 2016; **6:** 19121.

Table S1. *PIM1* and *NOTCH3* connection to overall survival in breast cancer. Kaplan-Meier analyses were performed to different subgroups of clinical breast cancer data at kmplot.com for *PIM1* and *NOTCH3* mRNA levels. Significant differences (P<0.05) are enhanced in bold.

**Subtype**	**Estrogen receptor status**	**Grade**	**Gene**	**Connection to poor survival**	**P-value**	**Patient number**
Any	Any	Any	*PIM1*	No	**0.0025**	3115
Any	Any	Any	*NOTCH3*	Yes	**0.037**	3115
Basal	Any	Any	*PIM1*	No	**0.042**	450
Basal	Any	Any	*NOTCH3*	ns	0.3	450
Her2	Any	Any	*PIM1*	ns	0.14	222
Her2	Any	Any	*NOTCH3*	Yes	**0.036**	222
Luminal A	Any	Any	*PIM1*	No	**0.017**	1524
Luminal A	Any	Any	*NOTCH3*	ns	0.27	1524
Luminal B	Any	Any	*PIM1*	No	**0.03**	919
Luminal B	Any	Any	*NOTCH3*	ns	0.23	919
Any	pos	Any	*PIM1*	No	**0.035**	1337
Any	pos	Any	*NOTCH3*	ns	0.058	1337
Any	neg	Any	*PIM1*	No	**0.016**	535
Any	neg	Any	*NOTCH3*	ns	0.11	535
Luminal A	pos	Any	*PIM1*	No	**0.044**	906
Luminal A	pos	Any	*NOTCH3*	ns	0.093	906
Luminal A	neg	Any	*PIM1*	No	**0.0092**	112
Luminal A	neg	Any	*NOTCH3*	ns	0.26	112
Any	pos	1	*PIM1*	ns	0.32	316
Any	pos	1	*NOTCH3*	ns	0.18	316
Any	pos	2	*PIM1*	ns	0.28	651
Any	pos	2	*NOTCH3*	ns	0.052	651
Any	pos	3	*PIM1*	Yes	**0.031**	356
Any	pos	3	*NOTCH3*	Yes	**0.0043**	356

Table S2: X-ray Data collection and refinement statistics.

Data Collection Statistics

**Resolution (Å)**	71.39 – 2.41
**Space Group**	$P2_12_12_1$
**Wavelength (Å)**	0.97919
**Unit Cell a, b, c (Å)**	66.45, 97.66, 104.62
**Unit Cell α, β, γ (°)**	90, 90, 90
**R_{merge}**	0.109 (0.583)
**I/σI**	9.7 (2.7)
**CC(½)**	0.994 (0.705)
**Completeness (%)**	99.4 (97.7)
**Redundancy**	5.5 (4.4)

() for highest resolution shell

Refinement Statistics

**R_{work}/R_{free} (%)**	19.88 / 23.34
**Number of reflections**	26,661
**Number of atoms**	4,131
**Complexes/asymmetric unit**	1
**Wilson B/Mean B value (Å^2)**	54.53 / 38.78
**RMSD Bond Lengths (Å)**	0.010
**RMSD Bond Angles (°)**	1.09
**Ramachandran (favored/outliers)**	97.42% / 0.25%

Table S3: Calorimetric binding data for NOTCH RAM domain peptides to CSL.

	$K\ (M^{-1})$	K_d (μM)	$\Delta G°$ (kcal/mol)	$\Delta H°$ (kcal/mol)	$-T\Delta S°$ (kcal/mol)
**N1 RAM**	$4.63 \pm 0.4 \times 10^{7}$	0.022	-10.46 ± 0.05	-12.71 ± 0.12	2.26 ± 0.11
**N3 RAM**	$5.49 \pm 0.9 \times 10^{6}$	0.187	-9.19 ± 0.1	-9.75 ± 0.17	0.56 ± 0.22
**N3 RAM pS1672**	NBD	---	---	---	---

All experiments were performed at 25°C. NBD represents no binding detected.

Table S4. Primers for site-directed mutagenesis of plasmid DNA. Mutagenesis of PIM target sites in the plasmid DNA encoding for Notch3 was performed with the following forward (F) and reverse (R) primers written in 5' to 3' direction.

Mutagenesis	Primer sequences
S1673A (SA)	F: CGAAAGCGAGAGCACGCGACCTTGTGGTTCCCAGAGGGTTTTGC<br>R: GCAAAACCCTCTGGGAACCACAAGGTCGCGTGCTCTCGCTTTCG
S2064A	F: CTGGGACCAAGAAGGCTAGAAGGCCACCCGGGAAGACCG<br>R: CGGTCTTCCCGGGTGGCCTTCTAGCCTTCTTGGTCCCAG
S1673E (SE)	F: GGCGAAAGCGAGAGCACGAGACACTATGGTTCCCTGAGG<br>R: CCTCAGGGAACCATAGTGTCTCGTGCTCTCGCTTTCGCC

Table S5. Guide RNA sequences for CRISPR/Cas9-mediated knock-out mutagenesis. Genomic DNA sequences encoding for NOTCH1 or NOTCH3 proteins in MCF-7 cells were knocked out by the CRISPR/Cas9 genome editing method using the following gRNA sequences (written in 5' to 3' direction).

Target gene	Target	gRNA sequence
NOTCH1	intronic region 5' – prime of exon 1	GGGGCTGGGACGCACACGCGCGG
NOTCH1	exon 2	GTGTGAAGCGGCCAATGGCACGG
NOTCH3	exon 33	GGGGCAGCCGGGCCCAATCG

Table S6. Design of the *NOTCH3 S1672A* targeting plasmid. *NOTCH3* exon 27 was targeted by gRNA 1, whilst gRNA 2 was designed to recruit Cas9 to exon 28. Coding sequences for those were obtained as a single synthetic gene block (gblock), which was ligated into a BbsI-digested pSpCas9(BB)-2A-GFP vector (PX458, Addgene; [9]). Each gRNA was expressed under a separate U6 promoter, with the promoter for gRNA 1 restored upon ligation of gblock to the hosting backbone and the promoter for gRNA 2 fully residing in the gblock. One extra guanine (G) was added in front of each gRNA for efficient expression. An additional third gRNA, targeting exon 27, was also designed and reserved as a back-up. All gRNA recognition sequences in the template plasmid were recoded, without altering amino acid sequences, to prevent Cas9 binding. Annotated sequences for described constructs are listed below. The protospacer adjacent motifs **(PAMs)** and BbsI sites are also highlighted.

Construct	***Exon target***	***Annotation***	***Sequence (5`-3`)***
gRNA1+ gRNA2 (gene block)	27, 28	Whole sequence	CCTTTTGGAAGACCTCACC**G**CCGCCGGCCCTTGTGACC AGGTTTTAGAGCTAGAAATAGCAAGTTAAAATAAGGCT AGTCCGTTATCAACTTGAAAAAGTGGCACCGAGTCGGT GCTTTTTTTCTAGACCCAGCTTTCTTGTTGTACAAAAA AGCAGGCTTTAAAGGAACCAATTCAGTCGACTGGATCC GGTACCAAGGTCGGGCAGGAAGAGGGCCTATTTCCCAT GATTCCTTCATATTTGCATATACGATACAAGGCTGTTA GAGAGATAATTAGAATTAATTTGACTGTAAACACAAAG ATATTAGTACAAAATACGTGACGTAGAAAGTAATAATT TCTTGGGTAGTTTGCAGTTTTAAAATTATGTTTTAAAA TGGACTATCATATGCTTACCGTAACTTGAAAGTATTTC GATTTCTTGGCTTTATATATCTTGTGGAAAGGACGAAA CACC**G**CAAGGGTGAGAGCCTGATGGGTTTGGGTCTTCG AGCTAG
gRNA1	27	gRNA	reverse-complement: **CC T**CT GGT CAC AAG GGC CGG CGG
		wild-type gDNA	G**CC T**CT GGT CAC AAG GGC CGG CGG GAA
		recoding	GC**T AG**C GGC CAT AAA GGG AGA AGG GAG
		protein	Ala-Ser-Gly-His-Lys-Gly-Arg-Arg-Glu
gRNA2	28	gRNA	C AAG GGT GAG AGC CTG ATG G**GG G**
		wild-type gDNA	GCC AAG GGT GAG AGC CTG ATG G**GG G**AG
		recoding	GCG AAA GGC GAA TCA CTC ATG G**GC G**AG
		protein	Ala-Lys-Gly-Glu-Ser-Leu-Met-Gly-Glu
gRNA3	27	gRNA	reverse-complement: **C CT**C TGG TTC CCT GAG GGC TTC
		wild-type gDNA	AC**C CT**C TGG TTC CCT GAG GGC TTC
		recoding	AC**A TT**G TGG TTT CCC GAA GGA TTT
		protein	Thr-Leu-Trp-Phe-Pro-Glu-Gly-Phe

Table S7. Assembly of the template plasmid for knock-in mutagenesis. For the template plasmid assembly, fragments 1, 3 and 4 were obtained separately, while fragments 2 and 5 were released by EcoRV digestion from one plasmid. Fragment content was the following: 1 contained the S1672A substitution; 2 contained loxP-flanked accessory parts for selection (Hygromycin resistance gene) and sorting (mCardinal fluorescent protein); 3 and 4 were amplified from *NOTCH3* (*N3,* NCBI gene ID: 4854) genomic DNA (gDNA) of WT MCF7 cells and intended to serve as 5' and 3' -end homology arms, facilitating transgene integration and homology-directed repair (HDR); 5 was the vector`s backbone and contained functional gRNA 1 and 2 recognition sites to improve the transgene release and to promote HDR. Shown are full sequences for the designed parts and partial sequences (three first and last nucleotides separated by dots) for published sequences. The DNA fragments were obtained from Integrated DNA Technologies (Coralville, USA).

Fragment number and sequence details	*Sequence (5`- 3`)*
**Fragment 1:** *N3* nucleotides 30305…30564, **S1672A mutation, recoded gRNA 3 recognition site,** *N3* nucleotides 30592…30665 (part of exon 26, whole exon 27), homology arms and sites *deleted* prior to Gibson Assembly.	*AGAAGCCCTCAGGGAACCAGAGG*ACC…CAC**GCTACATTGTGGTTTCCCGAAGGATTT**TCA…*GAAGAAGCCCTCAGGGAACCAGAGG*
**Fragment 2 (digested):** residuals of **EcoRV site**, homology arm, **PAM** and **recoded gRNA 1 recognition site,** *N3* nucleotides 30637…30783 (exon 27), **loxP site**, splice acceptor, **P2A linker** [13] **mCardinal** sites, cloning vector GloSensor(TM)-22F (GenBank: GU174434.1) nucleotides 4028…5999 (polyA, SV40 promoter, hygromycin resistance and polyA sites), **loxP** site, *N3* nucleotides 30784…30834, **recoded gRNA 2 recognition site**, **PAM,** *N3* nucleotides (part of exon 28 and homology arm) and residual **EcoRV site.**	**ATC**GGATTTTCACTGCACAAGGACGTGG**CTAGCGGCCATAAAGGGAGAAGGGAG**CCC…ACC**ATAACTTCGTATAGCATACATTATACGAAGTTAT**TGCTAACTACTAAACCACTGCCCATCCCCCCTTCTCTCTTCACAGGTTG**GGA…CCTATG…TAA,**ACT…GCA**,ATAACTTCGTATAGCATACATTATACGAAGTTATT**CTA…GGC**GAAAGGCGAATCACTCATGGGCG**AGGTGGCCACAGACTGGATGGACACAGAGTGCCCAGAGGCCAAGCGGCTAAAG**GAT**
**Fragment 3:** *N3* nucleotides 28348…30447 (exon 26 and part of exon 27). Homology arms are highlighted.	TATCCTGCTAGTGGCCTGGTAGAGGTACCAGAA…CCCTGCCTTGGGGAGGGGTGGCGGGGGCGGAGCTGGGGGCGGCCGAAGCCCCGCCTGAGGCCAAAGCCCCGCCCTCGGCTGAAGCCCCGCCCTCTGCTTCCTGCTCTTAGGGGAG
**Fragment 4:** *N3* nucleotides 30870…32525 (part of exon 28). Homology arms are highlighted.	ACTGGATGGACACAGAGTGCCCAGAGGCCAAGCGGCTAAAGGTA…AGCATTACTGAGGCACCCGCTGAATGTCTCTA
**Fragment 5 (digested):** residuals of **EcoRV sites**, homology arms, **PAMs**, **functional gRNA 1** (second site) **and 2** (first site) **recognition sites**, homing vector pUCIDT (Amp) from the nucleotide 483 through the plasmid back to the nucleotide 482 (ATC…GAT).	**ATC**ATTACTGAGGCACCCGCTGAATGTCTCTA**CCCCCATCAGGCTCTCACCCTTG**ATC…GAT**CGCCGGCCCTTGTGACCAGAGG**TATCCTGCTAGTGGCCTGGTAGAGGTACCA**GAT**

Table S8. Primers utilized in the knock-in mutagenesis. The primer pair 1 was used for validation of the targeting plasmid assembly. Pairs 2-4 were used for preparation of fragments 1, 2 and 4 of the template plasmid. Pairs 5-6 were used for validation of the template plasmid assembly (also in the screen of MCF7 clones, both after the transgene integration and Cre-mediated excision). Pairs 7-9 were used for validation of the template S1672A construct positioning in the genomic DNA. Pairs 10-11 were used for confirmation of the Cre recombinase-aided excision of the accessory parts of the template construct. The primers were obtained from Integrated DNA Technologies (Coralville, USA).

Pair	*Description of the PCR products*	*Primer sequences (5`-3`)*
**1**	1155 bp: gene block integration into the plasmid backbone<br>711 bp: empty circular backbone	**F:** TTTTTGTGATGCTCGTCAGG<br>**R:** GGGCGTACTTGGCATATGAT
**2**	fragment 1 (322 bp)	**F:** ACCCACTGCGGGACGTG<br>**R:** CCCTTGTGACCAGAGGCCA
**3**	fragment 2 (2099 bp)	**F:** TATCCTGCTAGTGGCCTGGT<br>**R:** CTCCCCTAAGAGCAGGAAGC
**4**	fragment 4 (1656 bp)	**F:** ACTGGATGGACACAGAGTGC<br>**R:** TAGAGACATTCAGCGGGTGC
**5**	3495 bp: correct fragment 3-1-2 assembly<br>639 bp: complete *Cre*-driven excision<br>3495 + 605 bp: unsuccessful nucleotide substitution	**F:** AATGATCACTGCTTCCCCGA<br>**R:** TCCTGGCGGACAATGGGAA
**6**	5193 bp: correct fragment 3-1-2-4 assembly	**F:** AATGATCACTGCTTCCCCGA<br>**R:** TAGAGACATTCAGCGGGTGC
**7**	3749 bp: correct integration of the left homology arm	**F:** GCAGGTTGCTAAGTGGCTTG<br>**R:** AAGTTAGTAGCTCCGCTTCCC
**8**	3167 bp: correct integration of the right homology arm	**F:** CTTCAGCTTCGATGTCGGC<br>**R:** ATCAGGATGACGCAGCCATT
**9**	3124 bp: correct integration of the right homology arm	**F:** CTTCAGCTTCGATGTCGGC<br>**R:** ATCAGGATGACGCAGCCATT
**10**	881 bp: removal of the accessory parts<br>847 bp: presence of a WT allele	**F:** ACCCACTGCGGGACGTG<br>**R:** GGAGGATTTTCCACCTGCCC
**11**	963 bp: removal of the accessory parts<br>926 bp: presence of a WT allele	**F:** AATGATCACTGCTTCCCCGA<br>**R:** GGAGGATTTTCCACCTGCCC

Table S9. Antibody dilutions and procedures used for Western blotting. Antibodies were ordered from Abcam (Cambridge, UK), Cell Signaling Technology (CST, Danvers, MA, USA), Sigma-Aldrich or Santa Cruz Biotechnology (Dallas, TX, USA). ECL™ Prime (GE Healthcare Life Sciences, Little Chalfont, UK) or Clarity Max™ (Bio-Rad Laboratories, Inc.) substrates were used for chemiluminescence, while signal intensities were analyzed by the ChemiDoc^{TM} MP Imaging System (Bio-Rad Laboratories, Inc., Hercules, CA, USA).

**Target**	**Dilution**	**Clone/ code**	**Manufacturer**
activated NOTCH1	1:1000	ab8925	Abcam
PIM1	1:10000	EP2645Y, ab7577	Abcam
PIM1	1:500	#2907	CST
PIM2	1:500	#4730	CST
PIM3	1:500	#4165	CST
full-length NOTCH3	1:1000	#2889S	CST
cleaved NOTCH3	1:1000	#C2211	CST
GFP	1:1000	#2956S	CST
anti-phospho Ser/Thr (RXXS*/T*)	1:1000	#9614	CST
ACTB	1:1000	#3700, #4970	CST
HA-tag	1:1000	H3663	Sigma-Aldrich
Flag	1:500	FI804	Sigma-Aldrich
ACTB	1:20 0000	D13K4803	Sigma-Aldrich
PIM1	1:500	12H8, sc-13513	Santa Cruz
PIM1	1:500	19F7	Santa Cruz

Table S10. Primers used for qPCR.

Gene	Forward primer (5' – 3')	Reverse primer (5' – 3')
HES1	GAAGCACCTCCGGAACCT	GTCACCTCGTTCATGCACTC
HEY1	TGGATCACCTGAAAATGCTG	CGAAATCCCAAACTCCGATA
NOTCH1	CGGGGCTAACAAAGATATGC	CACCTTGGCGGTCTCGTA
NOTCH3	GCCATGCTGATGTCAATGCT	CAGCCCAGTGTAAGGCTGAT
UBC	CACTTGGTCCTGCGCTTGA	CAATTGGGAATGCAACTTTAT

Figure S1

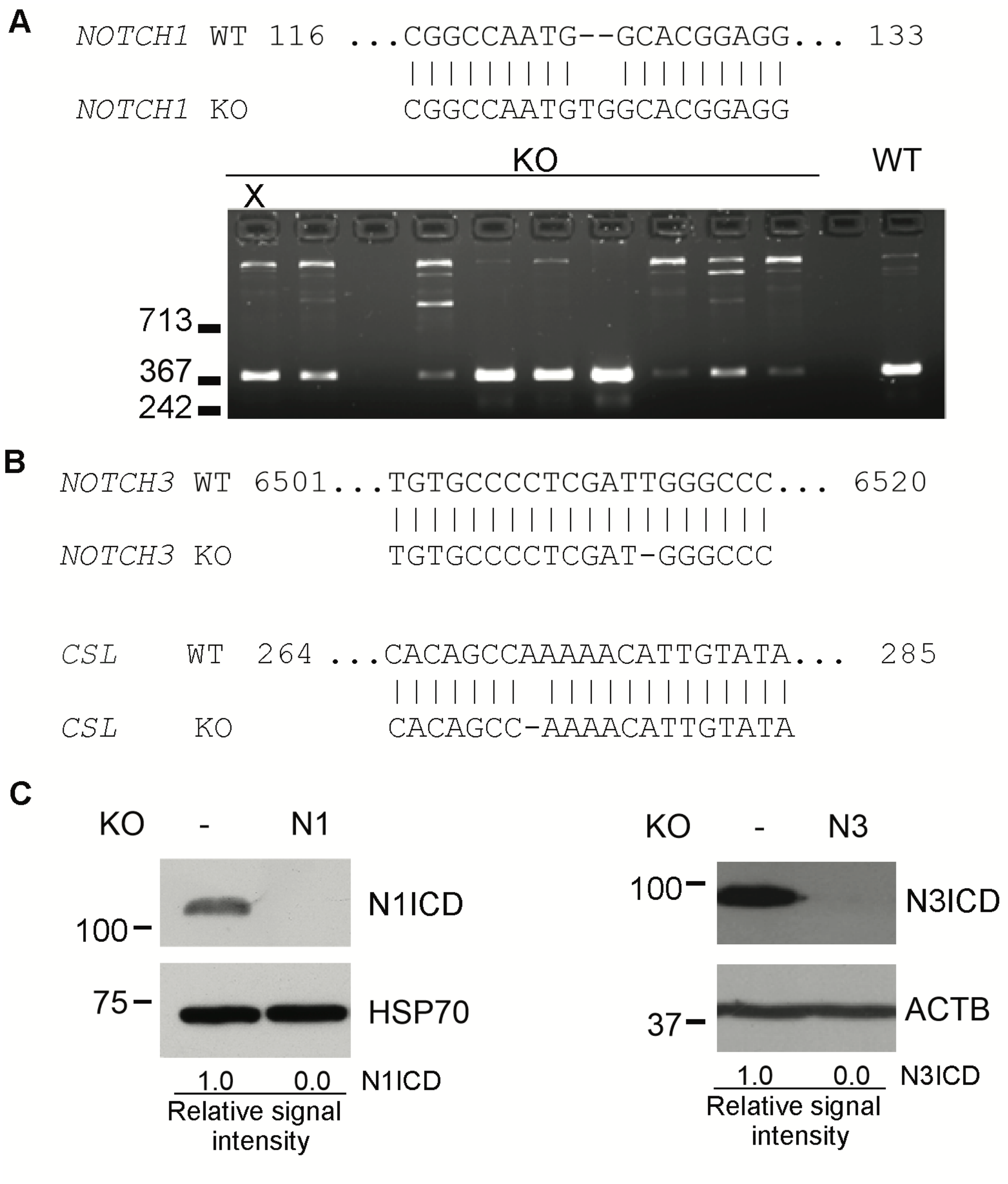

Figure S1. Verification of *NOTCH* and *CSL* knock-out cell lines. *NOTCH1* (N1), *NOTCH3* (N3) or *CSL* genes were knocked out from MCF-7 cells by the CRISPR/Cas9-based genome editing method. **A.** Insertion and reading frame shift of *NOTCH1* genomic DNA was confirmed by sequencing. PCR amplification was performed to identify any additional gene fragments as compared to the wild-type (WT) DNA. The chosen clone is marked by X. **B.** *NOTCH3* and *CSL* genomic DNA deletions were similarly confirmed by sequencing**. C.** Western blotting was used to confirm the absence of the N1ICD or N3ICD proteins in the MCF-7-based *NOTCH1*- or *NOTCH3*-deficient knock-out (KO) cells. HSP70 or ACTB stainings were used as loading controls.

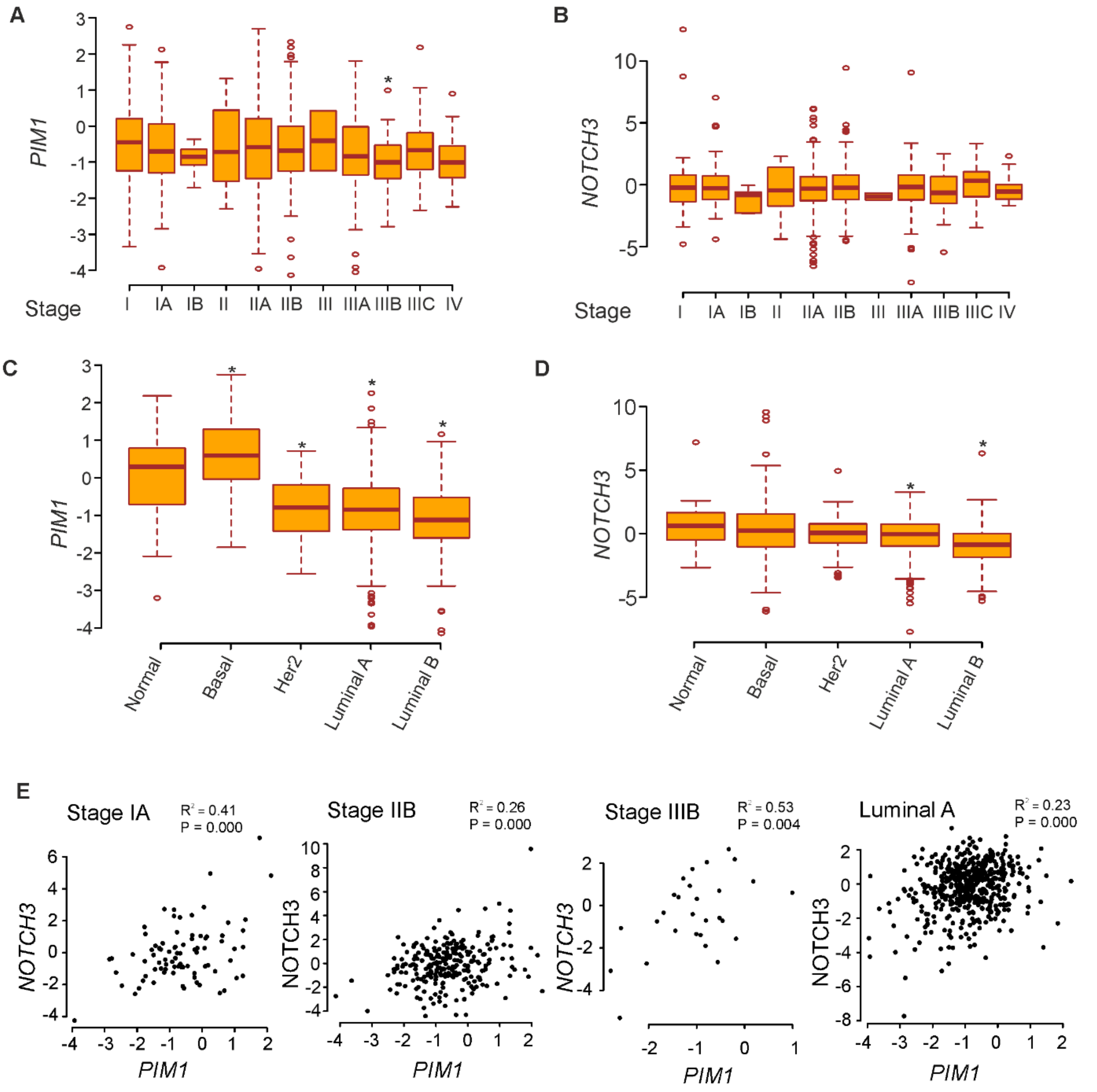

Figure S2. *PIM1* and *NOTCH3* expression correlates in breast cancer stages I-III and Luminal A subtype. A-D. *PIM1* and *NOTCH3* mRNA log2 fold changes were derived from the cBioPortal database, PanCancer Atlas dataset to visualize expression levels in clinical breast cancer. Shown are averages and standard deviations for each stage or subtype. **E.** Same datasets were used for analysis of gene expression correlations by Pearson correlation coefficiency (R^2). P<0.05 was considered as significant.

A RAM DOMAIN HOMOLOGY BETWEEN HUMAN NOTCH FAMILY MEMBERS

```
N1_1758 RKRRRQHGQLWFPEGFKV
N2_1700 AKRKRKHGSLWLPEGFTL
N3_1665 ARRKREHSTLWFPEGFSL
N4_1471 RRRRREHGALWLPPGFTR
```

ANK DOMAIN HOMOLOGY BETWEEN HUMAN NOTCH1 AND NOTCH3

```
                                                  α1                            α2        α3
N1_1872 MDVNVRGPDGFTTPLMIASCSGGGLETGNSEEEEDAP--AV-ISDFIYS---LHNQTDRTGETALHLAARYSRSDAAKRLLEASADAN
N3_1780 MDVNVRGPDGF-TPLMLASFCGGALEPMPTEEDEADDTSASIISDLICQGAQLGARTDRTGETALHLAARYARADAAKRLLDAGADTN
               α4         α5                          α6          α7                    α8
N1_1956 IQDNMGRTPLHAAVSADAQGVFQILIRNRATDLDARMHDGTTPLILAARLAVEGMLEDLINSHADVNAVDDLGKSALHWAAAVNNVDA
N3_1867 AQDHSGRTPLHTAVTADAQGVFQILIRNRSTDLDARMADGSTALILAARLAVEGMVEELIASHADVNAVDELGKSALHWAAAVNNVEA
         α9                  α10       α11                   α12       α13
N1_2044 AVVLLKNGANKDMQNNREETPLFLAAREGSYETAKVLLDHFANRDITDHMDRLPRDIAQERMHHDIVRLLDEYNLVRSPQLHG
N3_1956 TLALLKNGANKDMQDSKEETPLFLAAREGSYEAAKLLLDHFANREITDHLDRLPRDVAQERLHQDIVRLLDQPSGPRSPPGPH
```

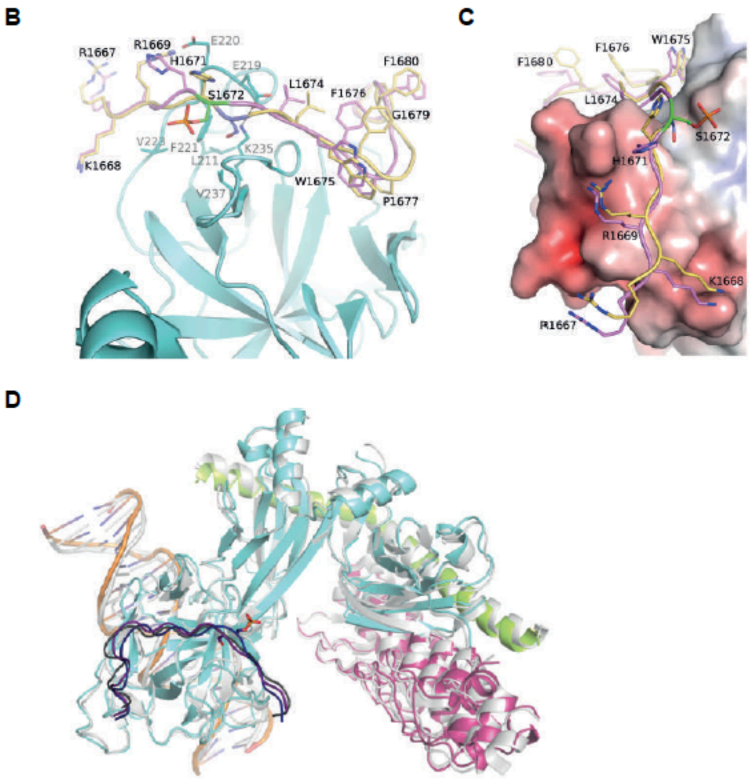

Figure S3. Model predicting the binding conformations of non-phosphorylated and phosphorylated NOTCH3 RAM to CSL. A. Homology comparisons of NOTCH RAM and ANK domains were done according to the BLAST: Basic Local Alignment Search Tool (NIH, NCBI) to human NOTCH1-4 (N1-4) amino acid sequences [14]. Compared residues are numbered. **B.** Modelling shows human Notch3 ICD non-phosphorylated RAM peptide (yellow) and S1672 (blue sticks) as well as the phosphorylated RAM peptide (pink) and S1672 (green sticks). CSL (cyan) consensus regions (yellow sticks) along with the hydrophobic cavity are lined by L211, F221, V223 and V237, and closed on one side by the salt bridge between E219 and K235 (black dashed line). **C.** Modelling of the charges of the RAM-binding area on CSL and the phosphate group on S1672 (positive charge in blue, negative charge in red and neutral areas in grey, color ranges from -7 to 7). RAM peptides are colored as in **B**. **D.** Comparison of non-phosphorylated NOTCH1 (grey) and NOTCH3 transcription complexes (RAM: purple, ANK: pink, CSL: cyan, MAML: green, DNA: orange sticks). N3ICD phosphorylation site S1672 missing from N1ICD is shown in sticks, while the displacement of the RAM peptide is shown in dark blue.

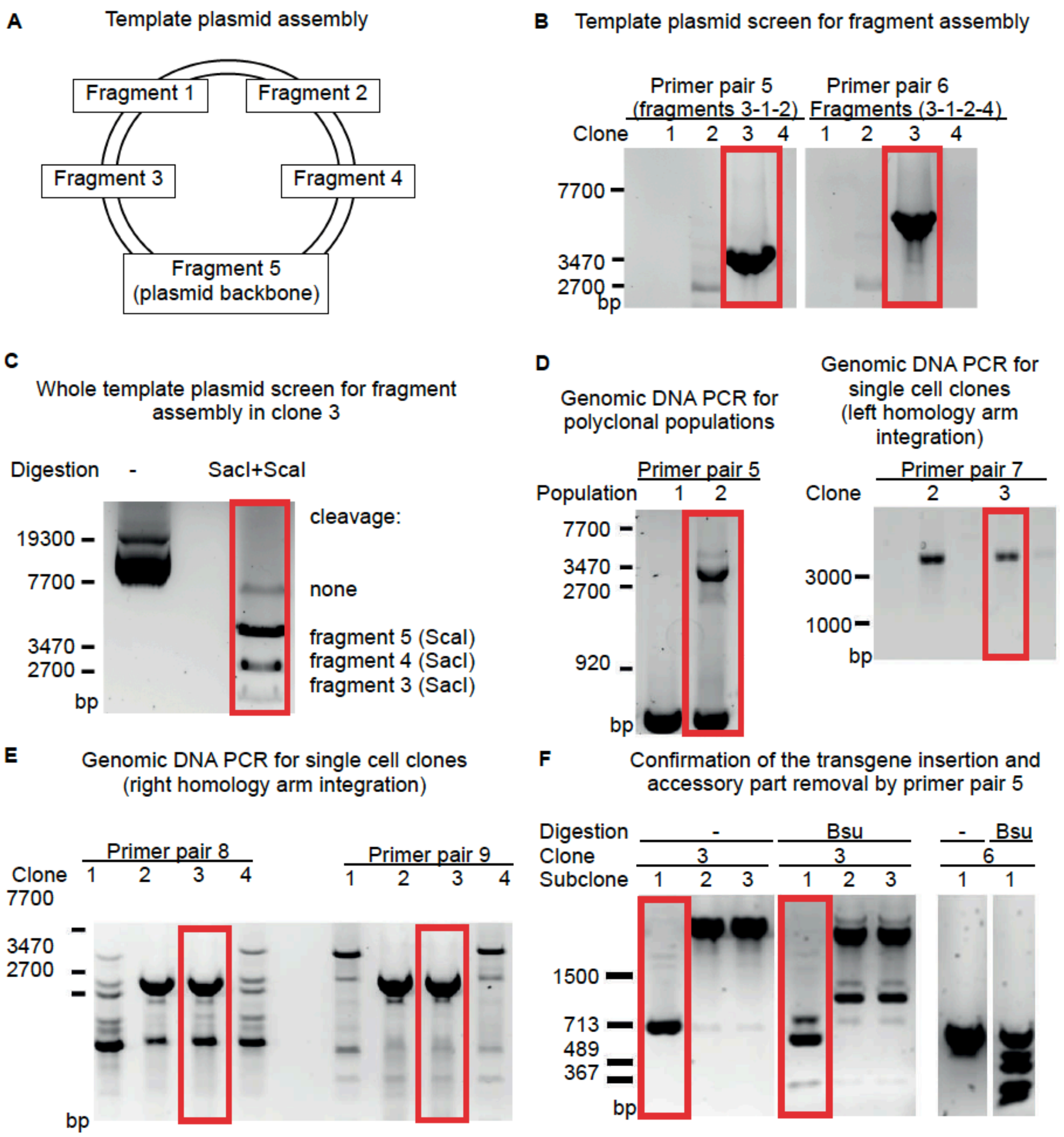

Figure S4. Validation of the template plasmid assembly for endogenous NOTCH3 S1672A knock-in mutagenesis. A. A schematic model of the template plasmid. **B.** PCR shows correct amplicons in bacterial clone 3. **C.** Double digestion of the plasmid-DNA indicates correct vector assembly. **D-E.** The polyclonal MCF-7 cell population 2 was used for single clone isolation (transgene 3495 bp, amplicons from WT *NOTCH3* 605 bp). Single cell -derived clones 2-3, further isolated from population 2, showed proper integration of the mutant transgene in the regions of both homology arms. **F.** The transgene insertion was confirmed by genomic DNA PCR and Bsu36I (Bsu) digestion, which led to 474/165 bp bands in the mutant subclone 3_1 and 246/194/165 bp bands in the negative control (subclone 6). The accessory part excision by the Cre/LoxP technique was confirmed by a 639 bp product as compared to the full length negative controls (3495 bp, clones 3_2 and 3_3), which also produced an additional 605 bp band from the wild type allele. Construct/clones chosen to establish the final cell line are lined with red.

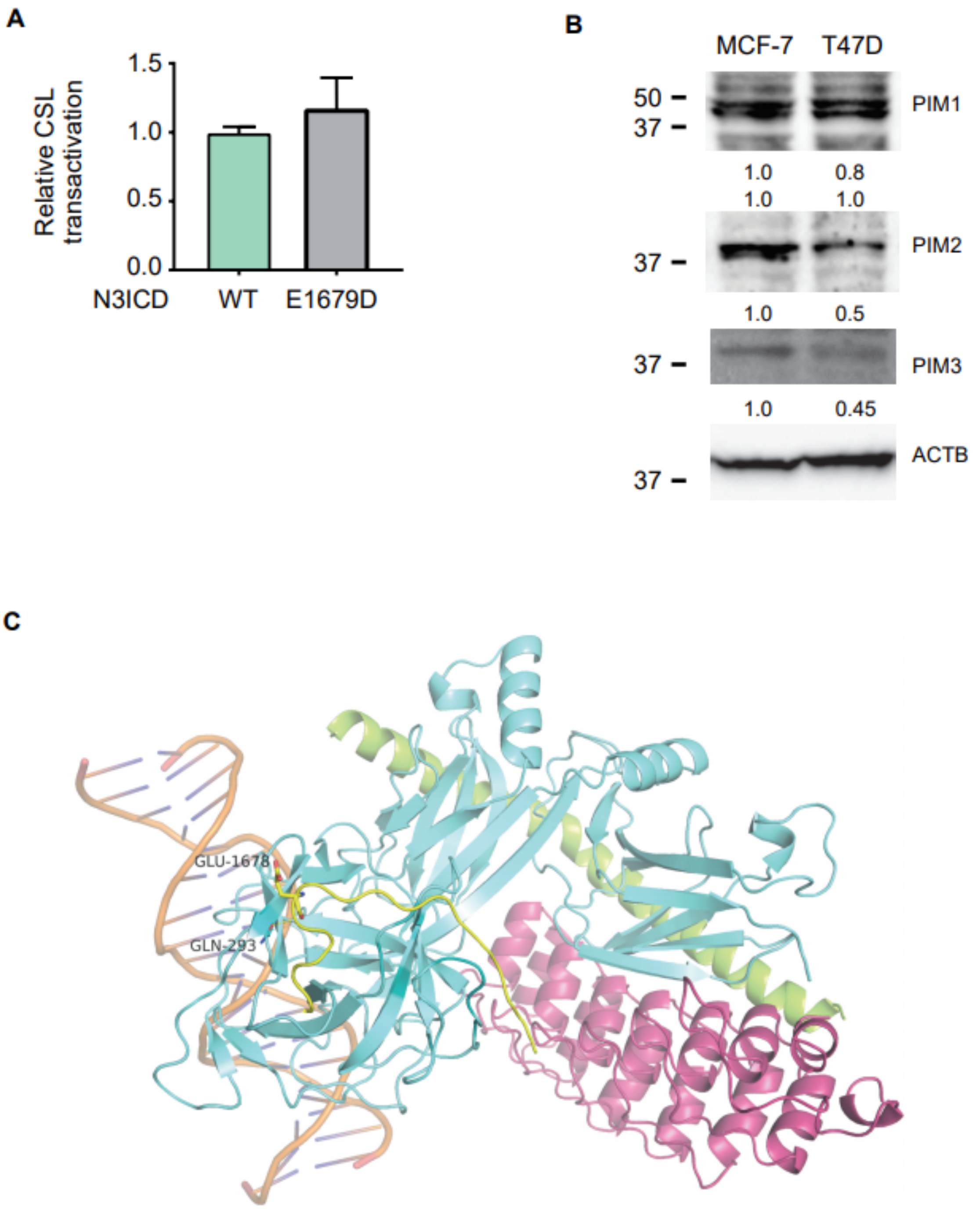

Figure S5. Characterization of the NOTCH3 E1678D mutation and cellular PIM expression levels. A. MCF-7 cells were transiently transfected to overexpress the mouse N3ICD E1679D mutant, which corresponds to the additional E1678D human mutation observed in the S1672A knock-in cell line. NOTCH3-mediated transactivation was measured by the 12xCSL-luciferase assay. Shown are average results from three parallel samples. **B.** Protein expression levels for PIM family members in MCF-7 and T47D cells. ACTB was used as a loading control. **C.** Human NOTCH3 E1678 is directed outwards from the interaction surface with CSL as shown by the molecular modelling.
